# Snapshot Redox Cycling Voltammetry (Snapshot RCV): Fast Generation-Collection Detection across a Wide Potential Range at a Chip-Based Microelectrode Array

**DOI:** 10.1021/acsmeasuresciau.6c00091

**Published:** 2026-07-22

**Authors:** Blake A. Sterling, Miguel Á. Ábrego Tello, Ingrid Fritsch

**Affiliations:** Department of Chemistry and Biochemistry, 3341 University of Arkansas, 345 N Campus Walk, Fayetteville, Arkansas 72701, United States

**Keywords:** chronoamperometric redox cycling voltammetry, microelectrode array, generation-collection electrochemistry, catecholamine detection, electron transfer kinetics, coupled chemical mechanisms, rapid analysis

## Abstract

During generation-collection, redox-active species oxidize (or reduce) at generator electrodes and electrochemically convert back at neighboring collector electrodes, yielding dual-current signals shaped by mass transport, reaction kinetics, and scaled by analyte concentration. As electrode spacing decreases, diffusion layers overlap, recycling species in a feedback loop (redox cycling, RC) and amplifying observed currents. While advantageous, conventional RC techniques experience challenges: chronoamperometry (CA) offers high temporal resolution but lacks thermodynamic insight, while cyclic voltammetry (CV) captures the potential window but is constrained by scan duration and background current at high speeds. Here, snapshot redox cycling voltammetry (Snapshot RCV) is introduced, which constructs the profile of a conventional RC voltammogram without a potential sweep. By simultaneously biasing individual generator electrodes in a microband array to different voltages across the potential range while individually holding collector electrodes at a reversing potential, high-resolution “spatial” voltammograms are obtained in as little as 1 s, providing steady-state generation-collection profiles on a chronoamperometric time scale. This method was evaluated using 0.50 mM Ru­(NH_3_)_6_Cl_3_ in 0.10 M KCl on a seven-electrode gold microband array (*width* = 4.08 μm, *gap* = 4.14 μm, *length* = 97.17 μm). Snapshot RCV was then performed on other analytes (dopamine (DA), 3,4-dihydroxyphenylalanine (L-DOPA), 3,4-dihydroxyphenylacetic acid (DOPAC), and ascorbic acid (AA)) with different electrochemical properties: standard reduction potentials (E°), electron transfer rate constants (k°), and following chemical reaction rates. Snapshot RCV captures the voltammetric shape with a 10%–60% increase in collection efficiency over Conventional RCV and can isolate inner array electrochemistry from edge contributions, allowing for postexperimental geometric filtering. The different collection efficiencies and voltammetric shapes from redox-active molecules, driven by their thermodynamics, kinetics, and chemical mechanisms (EC, ECC′), could make Snapshot RCV a promising technique for qualitative and quantitative analysis while enabling rapid sensing that is also adaptable to confined spaces.

## Graphical abstract

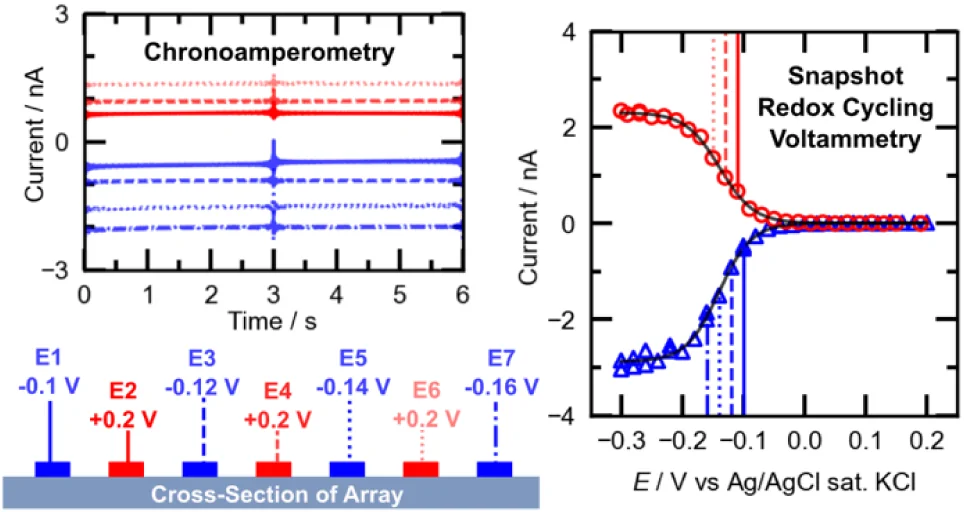

## 1 Introduction

This work introduces the electrochemical technique of snapshot redox cycling voltammetry (Snapshot RCV) to establish its feasibility as a generation-collection (GC) method and to prepare a foundation of understanding on which applications can be built. The approach exploits the spatial arrangement of an alternating generator–collector microelectrode array. Here, a potential set consists of a specific distribution of fixed potentials applied simultaneously across the array. Within each set, the generator electrodes (GEs) are held at different fixed potentials across the selected potential window, while the collector electrodes (CEs) between neighboring GEs are held at a potential that electrochemically converts the generated species back to its original form. The resulting currents are recorded simultaneously within each set. GE currents are plotted against their applied potentials, and each CE current is plotted against the average potential of its two neighboring GEs. Depending on the number of independently addressable electrodes and available potentiostat channels, the complete potential window can be sampled in one set or assembled from successive sets. Thus, generator and collector spatial voltammograms are constructed without a continuous potential sweep. Because the potential points within each set are measured in parallel, the response can be obtained on the time scale required for the generation-collection currents to develop rather than the time required to sweep the potential window. Snapshot RCV is examined here using five electroactive species: a model compound, hexaammineruthenium­(III) ([Ru­(NH_3_)_6_]^3 +^ ), and four biochemical species, dopamine (DA), 3,4-dihydroxyphenylalanine (L-DOPA), 3,4-dihydroxyphenylacetic acid (DOPAC), and ascorbic acid (AA). These compounds^3–13^ were selected because their established electrochemical behaviors provide different tests of how thermodynamics, electron-transfer kinetics, diffusion, and following chemistry affect the voltammetric profiles and collection efficiencies.

In single-electrode systems, generation-collection measures the current of a heterogeneous electron-transfer reaction and then quickly interrogates the generated product electrochemically before mass transport carries it away from the interface.^14^ The applied potential is swept, stepped, or pulsed to achieve this function. Approaches include cyclic voltammetry (CV), double-potential-step chronoamperometry (CA), and pulse techniques such as square-wave voltammetry (SWV). They come with tradeoffs across diverse fields. CV provides information on redox potentials and electron-transfer kinetics through one or more scans at different scan rates, and species generated during the forward scan can be interrogated during the reverse scan. At high scan rates, however, charging current becomes a significant component of the measured current, particularly in fast-scan cyclic voltammetry.^3^ Like CV, CA also informs about mass transport and heterogeneous electron transfer kinetics. A potential reversal probes the generated products through a double-potential-step, first to a generation potential and then to a collection potential.^15^ Time resolved measurements such as capturing transient arrivals of analytes are possible by continual monitoring at a fixed potential or quickly stepping between generating and collecting potentials. Unlike CV, the charging current can be minimized by sampling faradaic current past the cell time constant, but the potential must be determined in advance based on prior knowledge of the chemical system, limiting chemical specificity and discovery of unknown reactions and other species with different thermodynamics. SWV provides both potential-dependent information and diminished charging-current contributions, but its fixed pulse amplitude limits independent control of the generation and collection potentials.^16^ In each case, the collector response depends on the concentration profile established during the preceding generation step at the same electrode.

In contrast, spatially separated GC allows a generator electrode (GE) and collector electrode (CE) to operate simultaneously under independent potential control. One established example is the rotating ring–disk electrode (RRDE), which uses rotation to control mass transport between the two electrodes.^6,17–19^ Rotation continuously supplies fresh solution to the inner disk GE and transports the generated species outward to the surrounding ring CE. The ring measures the fraction of disk-generated species that reaches it and the flow carries the collected species away from the electrode assembly. This controlled hydrodynamic transport allows steady-state currents to be established and makes RRDEs useful for investigating diffusion coefficients, electron-transfer kinetics, catalysis, and coupled chemical reactions.

Scanning electrochemical microscopy (SECM) provides a diffusion-controlled form of spatially separated GC using a movable microelectrode tip positioned above a substrate.^20^ Species transport between the two interfaces by diffusion rather than directed convection. When the species generated at the tip diffuses to an electroactive substrate where it is converted back to its original form and then returns to the tip to react again, this is redox cycling (RC), producing positive feedback and causing the measured current to increase as the distance decreases. At an insulating or nonreactive substrate, diffusion of the redox species to the tip is shielded, producing negative feedback and causing the measured current to decrease as the distance decreases. The current response as a function of the tip–substrate gap is used to investigate heterogeneous electron-transfer kinetics (E) and mechanisms involving following chemical reactions (EC) and catalytic following reactions (EC′).^20–29^ However, varying this gap requires precise mechanical positioning of the tip during the measurement.^20,30^

An alternative is to establish a small, fixed distance between the GE and CE during device fabrication that can be integrated with different electrode geometries and spatial arrangements. Fixed-gap configurations include: dual-barrel electrodes,^31^ alternating conducting and insulating layers,^32–37^ and photolithographically fabricated electrodes.^38–42^ Coplanar microband (and nanoband) electrode arrays are a common configuration for RC and consist of two or more electrodes with alternating GEs and CEs, producing two electrochemical signals. Decreasing the interelectrode gap (*w*_*gap*_) increases the concentration gradients of generated and collected species, amplifying the RC currents. Increasing the number (*m*), length (*l*) and width (*w*) of the generator-collector electrode pairs also increases the RC currents.^2^

Equation 1 ^43^ and 2 (modification of eq 1)^44^ illustrate the relationship of an ideal limiting RC current, *i*_*lim*_ for a microband configuration:

$$i_{lim}=mnlFc^{*}D\left\{0.637\;ln\left[2.55\left(1+\frac{w}{w_{gap}}\right)\right]-0.19{\left(1+\frac{w}{w_{gap}}\right)}^{-2}\right\}$$ 1

$$i_{lim}=mnlFc^{*}D\left\{0.637\;ln\left[2.55\left(1+\frac{w}{w_{gap}}\right)\right]-0.19{\left(1+\frac{w}{w_{gap}}\right)}^{-2}+\frac{1.90h}{w_{gap}}\right\}$$ 2

In eqs 1 and 2, *w = w*_*GE*_ *= w*_*CE*_; *m = m*_*GE*_ *= m*_*CE*_; *n* is the number of moles of transferred electrons per mole of electroactive species; *l* is the length of the electrodes; *F* is the Faraday constant; c* is the total solution concentration of both forms of redox species; *D* is the diffusion coefficient for both the oxidized (O) and reduced (R) species (where *D*_O_ = *D*_R_). Equation 1 assumes that the electrodes are in-plane with the substrate. Equation 2 introduces 1.90­(*h*/*w*_*gap*_) to account for electroactive sidewalls (height, *h*). These equations make the following assumptions. The heterogeneous electron transfer reaction kinetics of the analyte, *k* ^0^, are fast, and thus, the current is mass transport limited by hemicylindrical diffusion. Contributions to diffusion from the outermost electrodes in the array (array edge effects), from the edges of the electrodes (electrode edge effects) and from the ends of the electrodes with a finite length (electrode end effects) are ignored. The number of GEs and CEs is sufficiently large such that there are no unique contributions from an uneven number of them. In addition, all species generated at GEs reach the adjacent CEs and remain electrochemically active with no loss to the bulk or conversion to products through following chemical reactions (*i*_*lim*_ *= i*_*lim,GE/RC*_ *= i*_*lim,CE*_).

Experimental redox-cycling behavior is usually evaluated from three current measurements: the generator current without redox cycling, *i*_*GE*_, the generator current during redox cycling, *i*_*GE/RC*_, and the simultaneously measured collector current, *i*_*CE*_. Species returning to the GEs from the CEs increases the generator current and this increase is represented by the amplification factor, *A*_*f*_ = *i*_*GE/RC*_/*i*_*GE*_. The fraction of generated species that arrive at the adjacent CEs is represented by the collection efficiency, *%C*_*e*_ = (100 × |*i*_*CE*_ */i*_*GE/RC*_|).

Collection efficiencies are typically less than 100% and can be used to understand redox mechanisms. For example, the *%C*_*e*_ obtained at interdigitated arrays (IDAs) of micro and nanoband electrodes with different interelectrode spacings have been used to investigate EC kinetics.^45^ This is possible because the *w*_*gap*_ across which molecules diffuse correlates to reaction time. IDAs consist of two interleaved combs, with one comb operating as the GEs and the other as the CEs.

Because collection efficiency depends on whether the generated species survives long enough to reach the collector, the same principle can be applied spatially within a single array. When the electrodes are individually addressable, selected GEs and CEs can be activated at different separation distances, and the current at each collector can be measured independently. This allows the survival of the generated species to be evaluated over different diffusion times without changing the device. This approach was used at an array with 4 μm wide electrodes and 4 μm wide gaps^46,47^ to differentiate dopamine (DA), norepinephrine (NE), and epinephrine (EP) in mixtures. They differ by their intramolecular cyclization rates of the orthoquinone forms produced at the GEs. Immediately adjacent CEs do not detect the orthoquinone of EP, because its following chemistry is too fast to survive the 4-μm trip. Similarly, CEs that are 28-μm away from the GEs can only detect the orthoquinone of DA, but not that of NE, whose following chemistry is faster than for DA.

Conventional redox cycling can be performed using either chronoamperometry or voltammetry. In the chronoamperometric approach, the GE potential is stepped past the standard reduction potential, E°, of the analyte, while the CEs are simultaneously poised at a reversing potential. Generator and collector currents are measured as a function of time. This approach offers high temporal resolution but lacks thermodynamic insight. Voltammetry at the GEs involves a linear potential sweep past E°, which can be optionally returned to the starting potential. The CEs are simultaneously poised at the starting potential. This Conventional RCV approach captures the current response across the potential window but is constrained by scan duration and background current at high speeds. Both approaches can determine analyte concentration from the current magnitude and following chemistry kinetics from the *%C*_*e*_. However, there is substantially more information built into the position and shape of the RCV response. The E_1/2_ reveals the thermodynamics of the redox couple and subsequent stabilization of its forms. The shape of the current–potential curve reflects the heterogeneous electron transfer kinetics.

Just as the dependence of *%C*_*e*_ on interelectrode spacing between GEs and CEs is unique for different redox species, so is the effect of the spacing on the shape of the RCV response.^2,48–50^ For example, we established that the steady state RCV response for a species with a moderately fast heterogeneous electron transfer rate (e.g., [Fe­(CN)_6_]^3–^) changes from a steep sigmoidal transition with a diffusion-limited plateau to a broader, shallower kinetically limited profile at a microband electrode array similar to the one investigated here.^2^ In that work, this transition was observed by decreasing w_gap_ from 4 to 0.6 μm. These specific effects of the interelectrode spacing on the collection efficiency and the RCV shape can be used to characterize electrochemical processes and may provide identifying features for chemical analysis.

The scan rate for Conventional RCV must be slow enough, however, to establish steady state between GEs and CEs while dynamically changing the potential. A scan rate at 0.020 V s^–1^, previously used at microelectrode arrays with the same dimensions as those employed here, requires 25 s to complete a 0.500 V potential window.

This required time scale can also be estimated from diffusion theory. To establish steady state redox cycling, diffusion lengths from adjacent GEs and CEs must completely overlap. This bidirectional coupling involves the diffusion of a redox species generated at a GE to a nearby CE, reconvert to the starting form, and diffuse back to the GE, simplistically viewed as a “round trip”. A diffusion length, δ, is equal to the one-dimensional root-mean square displacement of a collection of diffusing molecules for a given elapsed time *t*, *δ* = (2*Dt*)^1/2^.^14^ That length is *δ* = 6 μm for the center-to-center distance between adjacent GEs and CEs in an array with *w* = 4 μm and *w*_*gap*_ = 4 μm. Therefore, the time for two diffusion lengths to achieve the bidirectional coupling for a redox pair [Ru­(NH_3_)]^2+/3+^ is 2­(*δ* ^2^/(2*D*)) = 0.048 s, where we let *D* = *D*_*avg*_ = 7.45 × 10^–6^ cm^2^ s^–1^, the average of *D*_O_ = 7.1 × 10^–6^ cm^2^ /s^51^ and *D*_R_ = 7.8 × 10^–6^ cm^2^/s.^52^ In a linear potential sweep across 0.500 V with an applied potential resolution of 0.001 V, bidirectional coupling is completed 500 times for steady state redox cycling to be achieved across the entire voltammogram. The total time for this experiment would take 500 × 0.048 s = 24 s, further validating that a scan rate of 0.020 V/s is needed for Conventional RCV at an array of these dimensions. These long times can make chemical detection challenging when rapid analysis is needed, such as following the progress of chemical reactions,^53^ investigations of transient biological processes,^54,55^ and as a detector for chemical separations.^55–59^

To overcome this temporal limitation, we decouple the thermodynamic potential window from the time domain entirely. Rather than sweeping a common potential across the shorted GEs over time, Snapshot RCV distributes different fixed potentials simultaneously across the individually addressable GEs. By simultaneously biasing the individually addressable microbands to discrete, stepped potentials, we can perform parallel CA measurements. This approach effectively reconstructs the steady-state voltammetric profile of the analyte achieving the low-background, high-resolution benefits of a slow scan rate without the associated time penalties.

Here, the Snapshot RCV approach is investigated and compared to Conventional RCV. Seven electrodes in a microband array were employed, where *w* = 4.08 ± 0.24 μm, *w*_*gap*_ = 4.14 ± 0.29 μm, *l* = 97.17 ± 0.94 μm within an electroactive area of ∼70 μm × ∼100 μm (Figure 1). Multiple potential sets were used to reconstruct the Snapshot RCV profile. The resulting currents are compared to those predicted by eqs 1 and 2. We fit the current–potential curves to a sigmoidal model and quantitatively assess the response shapes for 0.50 mM concentrations of five different redox analytes with unique thermodynamics, kinetics and following chemistry. The ability of Snapshot RCV to isolate the behaviors of internal electrodes from those at edges of the array is also addressed. Based on these studies, possible applications for Snapshot RCV are discussed that would benefit from the technique’s speed and voltammetric capabilities.

**Figure 1:**
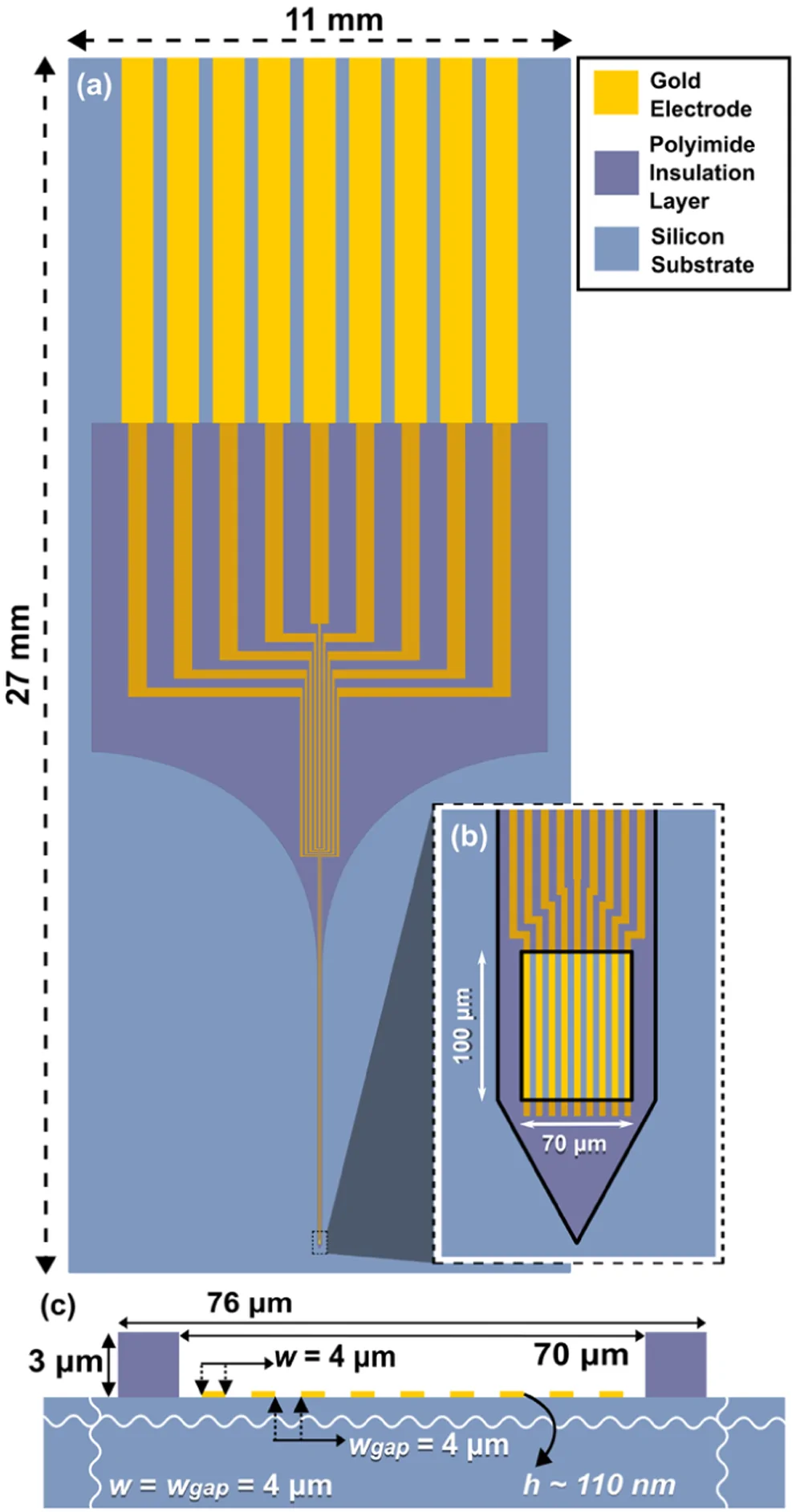
Drawings of (a) chip-based electrode device with contact pads and leads that taper to nine individual microband electrodes in an array, (b) expanded view of the array with 70 μm × 100 μm electroactive footprint in uninsulated rectangular area, and (c) cross-section of the electroactive area of the array on a silicon substrate (light blue) where white squiggly lines indicate that the silicon substrate extends beyond the scale of the diagram. The KMSF-1000 negative polyimide insulation layer is shown in dark purple. The heights of the electroactive sidewalls, *h =* ∼110 ± 5 nm, and the polyimide insulation layer (∼3 μm) are not drawn to scale. The insulation layer defines the length of the electroactive portion of the electrode array (∼100 μm). The probe-like shape of the insulation layer was designed for a previous application^1,2^ and is not relevant to the studies herein.

## 2 Methods and Materials

### 2.1 Chemicals and Materials

Unless otherwise stated, all chemicals were reagent grade and used without further purification. A chromium-plated tungsten rod and molybdenum boat from Kurt J. Lesker Company, Clairton, PA, USA and gold (99.99%) were used for thermal vapor deposition. Dopamine hydrochloride (DA), hexaammineruthenium­(III) chloride, L-(+)-ascorbic acid (AA), sodium dihydrogen phosphate monohydrate, 3,4-dihydroxyphenylalanine (L-DOPA), 3,4-dihydroxyphenylacetic acid (DOPAC) and 4-(2-methyl)-1-piperazineethanesulfonic acid (HEPES) (all ACS grade) were obtained from Alfa Aesar (Ward Hill, MA, USA). Magnesium sulfate was purchased from EM Science (Norwood, OH, USA). Dextrose was acquired from Fisher Scientific (Waltham, MA, USA). Ammonium hydroxide (50%), potassium chloride, and sodium chloride were obtained from Sigma-Aldrich Chemicals (St. Louis, MO, USA). Hydrogen peroxide (30%) was purchased from Avantor, previously VWR (Radnor, PA, USA). Aqueous solutions were made with water (ACS reagent grade ASTM type I, ASTM type II), obtained from Ricca Chemical (Arlington, TX, USA). The composition of artificial cerebrospinal fluid (aCSF) consisted of 100.0 mM NaCl, 5.0 mM KCl, 1.2 mM NaH_2_PO_4_, 5.0 mM NaHCO_3_, 10.0 mM dextrose, 2.5 mM HEPES, 1.2 mM MgSO_4_, 1.0 mM CaCl_2_ and adjusted to a pH of 7.4. AZ 1518 positive photoresist was obtained from Integrated Micro Materials (Argyle, TX, USA). KMSF 100 negative photoresist, KMSF 1000 developer, and Microposit 351 developer were acquired from Kayaku Advanced Materials, Inc. (Westborough, MA, USA). Acetone, isopropyl alcohol (IPA) and gold wet etchant were obtained from Fisher Scientific (Chicago, IL, USA). CR-7S chromium wet etchant was purchased from Capitol Scientific, Inc. (Austin, TX, USA). Nitrogen and oxygen gases used in microfabrication processes were obtained from Matheson Gas (Matheson, Topeka, KS), while argon and oxygen (both ultrahigh purity) for cleaning chips and purging solutions, respectively, were acquired from Airgas, Inc., (Airgas, Inc., Springdale, AR).

### 2.2 Microfabrication of Chip-Based Microelectrode Array Devices

Initial preparation of silicon wafers and thermal vapor deposition steps were performed at the University of Arkansas (Fayetteville, AR). The remaining microfabrication processes were performed at the KU Nanofabrication Facility (Lawrence, KS). Photolithographic processes including photoresist application and exposure were completed in an ISO class 5 cleanroom with amber lighting, while wet etching processes and wafer dicing were completed in ISO class 7 cleanrooms.

The 4-in. silicon (100) wafers (Silicon Quest International, Santa Clara, CA) with a 100-μm thick SiO_2_ layer were cleaned in a 5.4:0.6:1 (540.0 mL deionized water, 60.0 mL NH_4_OH (50%), 100.0 mL H_2_O_2_ (30%)) RCA cleaning solution for 10 min at 75.0 °C. Wafers were then submerged in a deionized water bath and rinsed three times under running deionized water, then dried under Ar gas. The cleaned wafers were then coated with chromium (∼8 nm), as an adhesion layer, and gold (∼ 100 nm) by thermal evaporation using an Edwards 306 Auto Thermal Evaporator (Edwards Electronics, Clinton, MA). The metal-coated wafers were plasma cleaned for 30 s under 10 cc/m^2^ flow O_2_ gas at 70 W of power using a PE-50 Plasma Reactor (Plasma Etch, Carson City, NV) before being exposed to hexamethyldisilazane (HMDS) in a Yield Engineering YES-310TA HMDS oven (YES Engineering Systems, Fremont, CA). AZ 1518 (∼1.5 μm) positive photoresist was spin-coated onto the wafers with a Brewer Science CEE 200CBX programmable spin coater (Cost Effective Equipment, St. James, MO) at 300 rpm for 10 s (100 rpm/s acceleration) followed by 3500 rpm for 20 s (300 rpm/s acceleration), and then soft-baked on a Brewer Science CEE 1300X hot plate (Cost Effective Equipment, St. James, MO) at 100 °C for 45 s. The coated wafers were then exposed to UV radiation through a chromium photopatterning mask (Advance Reproduction Corporation, North Andover, MA) in 2.0 s ± 0.1 s intervals (six times, 2 s on, 1 s off) for a total exposure time of 12.0 s at 14.2% intensity using an ABM UV flood source and OAI Mask Aligner system with a 365 nm UV LED and a ±0.1 s automatic shutter (ABM-USA, Inc., San Jose, CA; OAI, Inc., Milpitas, CA). After the exposure, the photoresist was developed for 10 s in Microposit 351 developer (1:2.5 with deionized water) with mild agitation before rinsing with deionized water and dried under N_2_.

The metals layers were then etched, first with gold wet etchant for 8 s with mild agitation, followed by rinsing with deionized water, and then with chromium wet etchant for 8 s with mild agitation before being rinsed thoroughly with deionized water several times, dried with N_2_, and vacuum baked (YES-310TA, YES Engineering Systems, Fremont, CA) for 30 min. During the vacuum baking, a warmup to 150 °C for 5 min was followed by a dehydration cycle (100 torr to 500 torr 3 times), and baked at 100 torr for 600 s. Finally, a purge cycle (10 torr to 500 torr 1 time) was followed by venting to atmosphere. No N_2_ was used during the process. Negative KMSF 1000 polyimide (∼3 μm) was spin-coated onto the wafers at 500 rpm for 10 s (500 rpm/s acceleration) followed by 4000 rpm for 30 s (500 rpm/s acceleration) and subsequently soft baked for 3 min at 110 °C. Wafers were then photopatterned through a chromium insulation mask and an exposure dose of 400 mJ/cm^2^ at 14.2% intensity before developing in KMSF 1000 developer (undiluted) for 60 s with mild agitation, rinsed with deionized water several times, dried with N_2_, and hard baked for 1 h at 175 °C. A sacrificial layer of AZ 1518 (∼1.5 μm) photoresist was spin-coated onto the wafers at 300 rpm for 10 s (100 rpm/s acceleration) followed by 3500 rpm for 20 s (300 rpm/s acceleration), and was used to protect the exposed electrodes from the water jet during dicing, (Disco DAD3221 Dicing Saw, Disco, Tokyo, Japan), into individual chips and subsequently removed using Microposit 351 developer (diluted to 1:2.5 with deionized water) for 20 s with rapid agitation after being exposed for 10 s to the UV flood source. The diced wafers were then rinsed thoroughly and sequentially with acetone, IPA, and deionized water, and then dried with N_2_ before storing in air in an enclosed container.

Microfabrication processes produced 12 chip-based devices on each wafer. Figure S1 of the Supporting Information shows an image of one of these completed chips. Inspection of the completed devices revealed nine well-defined 4.08 ± 0.24 μm wide planar microband electrodes (*N* = 9), *w*, with 4.14 ± 0.29 μm wide gaps, *w*_*gap*_, (*N* = 8), 97.17 ± 0.94 μm in length, *l*, (*N* = 9), within an electroactive window, 70 μm × 100 μm, of a negative polyimide insulation layer. An image of the microband array taken with optical microscopy is provided in Figure S2 of the Supporting Information. Device measurements were obtained using ImageJ image processing software from microscopy images of the device array. Adhesion of the insulation layer remained unaffected by both storage in air and deionized water for extended periods of time (longer than four months) as apparent by the lack of bubble-like patterns between the metal electrodes and the insulation layer during visual inspection. Before the devices were used for electrochemical experiments, they were rinsed with deionized water, dried with Ar, and cleaned of residual organic residues on the electrode surfaces by oxygen plasma (6.8 W applied to RF coil for 5 min at 60 mTorr, PDC-32G, Harrick Plasma, Ithaca, NY, USA) and stored in deionized water until use.

### 2.3 Electrochemical Characterization of Chip-Based Microelectrode Array Devices

#### 2.3.1 Electrochemical Setup

All electrochemical experiments were performed with an 8-channel potentiostat (1030A, CH Instruments, Austin, TX) equipped with a Faraday cage (CH Instruments, Austin, TX). The reference and counter electrodes were Ag/AgCl (saturated KCl) and platinum flag, respectively. A Sullins dual female edge connector (Sullins Connector Solutions, part number: RBE 10DHHN) with a 1.0 mm pitch and 10 contacts was used to connect the device to the potentiostat. Aqueous 0.50 mM Ru­(NH_3_)_6_Cl_3_ in 0.10 M KCl was prepared beforehand, purged with Ar for at least 15 min before experiments, and stored in a refrigerator at 12 °C between uses. Solutions containing 0.50 mM DA, 0.50 mM L-DOPA, 0.50 mM DOPAC, or 0.50 mM AA in aCSF were prepared by first making a 5.0 mM solution in aCSF and diluting to 0.50 mM with aCSF and used within 2 min. The aCSF was purged for at least 15 min before solutions were prepared.

#### 2.3.2 Electrode Designations and Electrode Configurations

Figure 1 shows a schematic of the nine electrodes in the device array. The device design was reported previously for use in other studies.^1,2^ A subset of seven adjacent electrodes was chosen for conventional and snapshot redox cycling studies. The number of electrodes was limited by the number of channels available from the potentiostat and by the chosen electrode configuration involving an odd number of electrodes where both flanking electrodes served as generators. The electrodes that exhibited the most similar faradaic currents for a particular analyte when characterized individually by CV in [Ru­(NH_3_)_6_]^3+^ were used for a given redox cycling experiment. In all discussions in this manuscript, the electrodes in the seven-electrode subset are always designated as E1 through E7, regardless of which seven adjacent electrodes were used in the nine-electrode array from Figure 1. The quasi-steady state currents obtained from CV characterization responses are referred to as *i*_*E1*_, *i*_*E2*_, *i*_*E3*_, *i*_*E4*_, *i*_*E5*_, *i*_*E6*_, and *i*_*E7*_, respectively.

In all redox cycling studies, electrodes 1, 3, 5, and 7 served as generator electrodes (GEs) and electrodes 2, 4, and 6 served as collector electrodes (CEs). For Conventional RCV, a potential sweep was applied at the GEs that were shorted together (E_1,3,5,7_) while a constant potential was held at the CEs, which were also shorted together (E_2,4,6_). The current for the combined GEs is *i*_*GE/RC,1,3,5,7*_, and that for the combined CEs is *i*_*CE,2,4,6*_. When the CEs are at open circuit, i.e., there is no redox cycling, the current at the combined GEs is *i*_*GE,1,3,5,7*_.

For Snapshot RCV, the current at each electrode was monitored individually and separately when different constant potentials were applied to individual GEs and a single constant potential was applied to the individual CEs. Currents at each of the GEs are designated as *i*_*GE/RC,En*_, where *n* is the number designation of the electrode in the seven-electrode subset array. Likewise, the currents at individual CE electrodes are designated as *i*_*CE,En*_.

The IUPAC sign convention for current is used for all figures (i.e., the cathodic current is negative). Comparisons of electrochemical responses within the text refer to the absolute values of the current magnitudes.

#### 2.3.3 CV Characterization of Individual Electrodes with a Model Compound

Electrodes E1 through E7 were characterized using CV with a model redox compound, 0.50 mM [Ru­(NH_3_)_6_]^3+^ in 0.10 M KCl, to establish electrochemical performance and to compare with expected background-subtracted faradaic current from approximate equations. CV responses were obtained at 0.020 V·s^–1^ from +0.200 V to −0.300 V and back to +0.200 V vs Ag/AgCl (saturated KCl). The background-subtracted faradaic currents were obtained by subtracting the currents in 0.10 M KCl alone from those in [Ru­(NH_3_)_6_]^3+^ in 0.10 M KCl at 0.001 V across the entire potential range.

#### 2.3.4 Redox Cycling Voltammetry with a Model Compound

Conventional RCV studies were performed with in the solution containing 0.50 mM [Ru­(NH_3_)_6_]^3+^. A potential sweep of 0.020 V·s^–1^ from +0.200 V to −0.300 V and back to +0.200 V vs Ag/AgCl (saturated KCl) was performed on the shorted GEs while CEs were held at open circuit. Then, the same potential sweep was performed on the shorted GEs while a constant anodic potential of + 0.200 V was held at the shorted CEs. The current responses, *i*_*GE,1,3,5,7*_, *i*_*GE/RC,1,3,5,7*_, and *i*_*CE,2,4,6*_, were plotted as a function of the generator potential.

Snapshot RCV studies were performed on the same seven-electrode subset array as Conventional RCV experiments with the same model compound solution. Chronoamperometry was used to monitor the current responses from individual electrodes, and a unique potential was held at each GE while a reversing potential for the analyte of interest was held at the CEs. Experiments were performed in multiple sets to provide more measurements of current along the potential axis while spanning the same potential range as Conventional RCV. Potentials at each electrode were individually controlled. The potentials were applied simultaneously and held for a 1 s quiet time, before recording the current for a subsequent 6 s. GEs were held at separate potentials increasing by either 0.060 or 0.020 V per generator. Between sets, each generator was held at a potential incremented by 0.060 V from the electrode’s designated potential in the previous set. There was a 5.8 ± 0.2 s delay between sets for the data from the previous set to be saved. Sets are listed in Table 1. For 0.50 mM [Ru­(NH_3_)_6_]^3+^, E1 was held constant at −0.060 V, while E3, E5, and E7 were held constant at −0.120 V, −0.180 V, and −0.240 V, respectively for set one. In set two, E1 was held constant at +0.200 V while E3, E5, and E7 were held constant at +0.180 V, +0.160 V, and +0.140 V, respectively. Between set two and set ten, individual GEs were cathodically incremented by −0.060 V per set. CEs were individually held constant and monitored at +0.200 V regardless of the set.

**Table 1:** Generator and Collector Potentials per Set in Snapshot-RCV for Different Solutions

Set Number	Generator E1	*Collector E2*	Generator E3	*Collector E4*	Generator E5	*Collector E6*	Generator E7
**0.50 mM [Ru(NH**_**3**_)_**6**_]^**3+**^ **in 0.10 M KCl**							
**Set 1** ^a^	–0.060 V	*+0.200 V*	–0.120 V	*+0.200 V*	–0.180 V	*+0.200 V*	–0.240 V
**Set 2** ^b^	+0.200 V	*+0.200 V*	+0.180 V	*+0.200 V*	+0.160 V	*+0.200 V*	+0.140 V
**Set 3** ^b^	+0.140 V	*+0.200 V*	+0.120 V	*+0.200 V*	+0.100 V	*+0.200 V*	+0.080 V
**Set 4** ^b^	+0.080 V	*+0.200 V*	+0.060 V	*+0.200 V*	+0.040 V	*+0.200 V*	+0.020 V
**Set 5** ^b^	+0.020 V	*+0.200 V*	+0.000 V	*+0.200 V*	–0.020 V	*+0.200 V*	–0.040 V
**Set 6** ^b^	–0.040 V	+0.200 V	–0.060 V	+0.200 V	–0.080 V	+0.200 V	–0.100 V
**Set 7** ^b^	–0.100 V	*+0.200 V*	–0.120 V	*+0.200 V*	–0.140 V	*+0.200 V*	–0.160 V
**Set 8** ^b^	–0.160 V	*+0.200 V*	–0.180 V	*+0.200 V*	–0.200 V	*+0.200 V*	–0.220 V
**Set 9** ^b^	–0.220 V	*+0.200 V*	–0.240 V	*+0.200 V*	–0.260 V	*+0.200 V*	–0.280 V
**Set 10** ^b^	–0.260 V	*+0.200 V*	–0.280 V	*+0.200 V*	–0.300 V	*+0.200 V*	–0.300 V
**0.50 mM DA**, L-DOPA, or DOPAC in aCSF							
**Set 11** ^a^	+0.180 V	*–0.100 V*	+0.240 V	*–0.100 V*	+0.300 V	*–0.100 V*	+0.360 V
**Set 12** ^b^	–0.100 V	*–0.100 V*	–0.080 V	*–0.100 V*	–0.060 V	*–0.100 V*	–0.040 V
**Set 13** ^b^	–0.040 V	*–0.100 V*	–0.020 V	*–0.100 V*	+0.000 V	*–0.100 V*	+0.020 V
**Set 14** ^b^	+0.020 V	–0.100 V	+0.040 V	–0.100 V	+0.060 V	–0.100 V	+0.080 V
**Set 15** ^b^	+0.080 V	*–0.100 V*	+0.100 V	*–0.100 V*	+0.120 V	*–0.100 V*	+0.140 V
**Set 16** ^b^	+0.140 V	*–0.100 V*	+0.160 V	*–0.100 V*	+0.180 V	*–0.100 V*	+0.200 V
**Set 17** ^b^	+0.200 V	*–0.100 V*	+0.220 V	*–0.100 V*	+0.240 V	*–0.100 V*	+0.260 V
**Set 18** ^b^	+0.260 V	*–0.100 V*	+0.280 V	*–0.100 V*	+0.300 V	*–0.100 V*	+0.320 V
**Set 19** ^b^	+0.320 V	*–0.100 V*	+0.340 V	*–0.100 V*	+0.360 V	*–0.100 V*	+0.380 V
**Set 20** ^b^	+0.380 V	*–0.100 V*	+0.400 V	*–0.100 V*	+0.420 V	*–0.100 V*	+0.440 V
**Set 21** ^b^	+0.440 V	*–0.100 V*	+0.460 V	*–0.100 V*	+0.480 V	*–0.100 V*	+0.500 V
**Set 22** ^b^	+0.500 V	*–0.100 V*	+0.520 V	*–0.100 V*	+0.540 V	*–0.100 V*	+0.560 V

a Corresponds to a change in 0.060 V per generator.

b Corresponds to a change of 0.020 V per generator.

The CA currents obtained in the Snapshot experiments were first smoothed using a Fourier transform algorithm with a cutoff of 50 Hz. Currents were then averaged over a 1 s interval between 4 and 5 s (0.002 s increments, *N* = 500) and plotted as a function of the generator potential. CE currents were processed in the same fashion and plotted as a function of the average adjacent generator electrode potentials. Sigmoidal fits of the GE and CE currents were performed for each redox species. Sampling the current between 4 and 5 s for the studies reported here was a conservative choice, well away from instrumental artifacts that were not always consistent, some of which appeared in the segment before 3 s (e.g., raw CA responses for sets 1 in Figures S4 and S7 in Supporting Information). Because the charging current contribution to the CA response is over within milliseconds, sampling the faradaic current with less noisy and more stable instrumentation in less than 1 s after the potential step should be possible and provide similar Snapshot RCV results as those reported here.

#### 2.3.5 Redox Cycling with Dopamine, L-DOPA, DOPAC, and Ascorbic Acid

Similarly, conventional redox cycling studies were performed with 0.50 mM dopamine, 0.50 mM L-DOPA, 0.50 mM DOPAC, and 0.50 mM ascorbic acid in aCSF solution using CV in a two-step process. A potential sweep of 0.020 V·s^–1^ from −0.100 V to +0.550 V and back to −0.100 V vs Ag/AgCl (saturated KCl) was performed on the shorted GEs while CEs were held at open circuit. Then, the same potential sweep was performed on the shorted GEs while a constant cathodic potential of −0.100 V was held at the shorted CEs. The current responses, *i*_*GE,E1,3,5,7*_, *i*_*GE/RC,E1,3,5,7*_, and *i*_*CE,E2,4,6*_, were plotted as a function of the generator potential.

Snapshot RCV studies were performed immediately following the Conventional RCV experiments using the same analyte solution. Chronoamperometry is used to monitor the individual electrodes, and a unique potential is held at each GE while a reversing potential for the analyte of interest is held at the individual CEs. Experiments were performed in sequential sets due to the limited number of channels available with the instrument. Potentials at each electrode were individually controlled and held constant for 6 s. GEs were held at separate potentials increasing by either 0.060 or 0.020 V per generator within sets. Between sets, each generator was held at a potential incremented by 0.060 or 0.080 V from the electrode’s potential in the previous set. Sets are listed in Table 1. For 0.50 mM dopamine, L-DOPA, DOPAC, or ascorbic acid in aCSF solution, E1 was held constant at an anodic potential of +0.180 V, while E3, E5, and E7 were held constant at +0.240 V, +0.300 V, and +0.360 V, respectively in set one. In set two, E1 was held constant at a cathodic potential of −0.100 V, while E3, E5, and E7 were held constant at −0.080 V, −0.060 V, and −0.040 V, respectively. Between set two and set twelve, GEs were incremented by 0.060 V anodically from the electrode’s potential in the previous set. CEs were individually held constant and monitored at −0.100 V regardless of set. The GE and CE currents for Snapshot RCV experiments were collected, processed, and plotted in the same manner as those obtained for 0.50 mM [Ru­(NH_3_)_6_]^3+^.

## 3 Results and Discussion

### 3.1 CV at Individual Electrodes and Conventional RCV at the Array Using a Model Compound

Figure S3 of the Supporting Information shows CV characterization at 0.020 V·s^–1^ of individual electrodes in the array in 0.50 mM [Ru­(NH_3_)_6_]^3+^ that was performed both before and at the conclusion of all the Conventional and Snapshot RCV studies reported in this manuscript. The sigmoidal shapes of the CV responses are as expected for microband electrodes as the potential sweeps toward potentials more negative than the measured *E*_*1/2*_ of −0.126 V vs Ag/AgCl (saturated KCl), achieving a quasi-steady state diffusion limited current before the sweep reverses direction. The magnitudes of the plateau currents at the electrodes are similar to each other and to that predicted by approximate equations derived for diffusion-limited current at hemicylindrical electrodes (eqs S1 and S2 in the Supporting Information). Consequently, quantitative comparisons of electrochemical behavior for experiments performed with the microelectrode array can be made with confidence. A more detailed discussion is provided in the Supporting Information.

Figure 2a shows the Conventional RCV responses at the seven-electrode array both with and without activating the CEs and in the same solution as that for CV of individual electrodes. First, E1, E3, E5, and E7 were shorted as the GEs and swept at 0.020 V s^–1^ from +0.200 V to −0.300 V and back, while E2, E4, and E6 were left at open circuit. The shape of the GE response is somewhat less sigmoidal than that for the individual electrodes, exhibiting a broadened peak in the plateau region, and a magnitude of the current in the forward sweep from −0.250 V to −0.300 V is 5.82 ± 0.02 nA that is only a factor of 0.61× the sum of the individual diffusion-limited, faradaic currents of the four electrodes of 9.58 ± 0.02 nA. When the GEs are shorted together and their potential swept at the slow scan rate employed, there is sufficient time for their diffusion layers to overlap, resulting in a shielding effect that explains the shape and magnitude of the shorted-GE CV response. A diffusion length of δ = 94.9 μm extends well beyond the width (*w*) and gap (*w*_*gap*_) of the electrodes, supporting the shielding effect explanation, where the time, *t,* is 6.05 s (taken as the time for the potential to sweep from *E_1/2_* to the potential where the current is measured in the plateau), and the averaged diffusion coefficient, *D*_*avg*_ = 7.45 × 10^–6^ cm^2^·s^–1^.^51,52^ (Diffusion coefficients for [Ru­(NH_3_)_6_]^3+/2+^ vary widely in the literature (6.4 × 10^–6^ cm^2^·s^–1^ to 9.4 × 10^–6^ cm^2^·s^–1^).^10^) When the same experiment is performed with the GEs, but E2, E4, and E6 serve as CEs and are simultaneously held at a constant potential of +0.200 V to oxidize the generated species [Ru­(NH_3_)_6_]^2+^ back to [Ru­(NH_3_)_6_]^3+^, redox cycling takes place. The Conventional RCV current in the forward sweep of the GEs is amplified to 10.68 ± 0.04 nA by a factor of *A*_*f*_ = 1.84 ± 0.05. Recycling the species increases the concentration gradient of [Ru­(NH_3_)_6_]^3+^ and therefore the flux to the GEs from the CEs, resulting in the amplified GE current. As long as the diffusional flux outpaces the kinetics, the CE current plotted as a function of the GE potential will have a sigmoidal shape. The standard heterogeneous electron transfer rate constant for [Ru­(NH_3_)_6_]^3+/2+^ is sufficiently fast that we would not expect it to distort the sigmoidal shape for the gap dimensions of the array.^10^ The magnitude of the CE current across its relatively flat plateau is 6.65 ± 0.05 nA.

**Figure 2:**
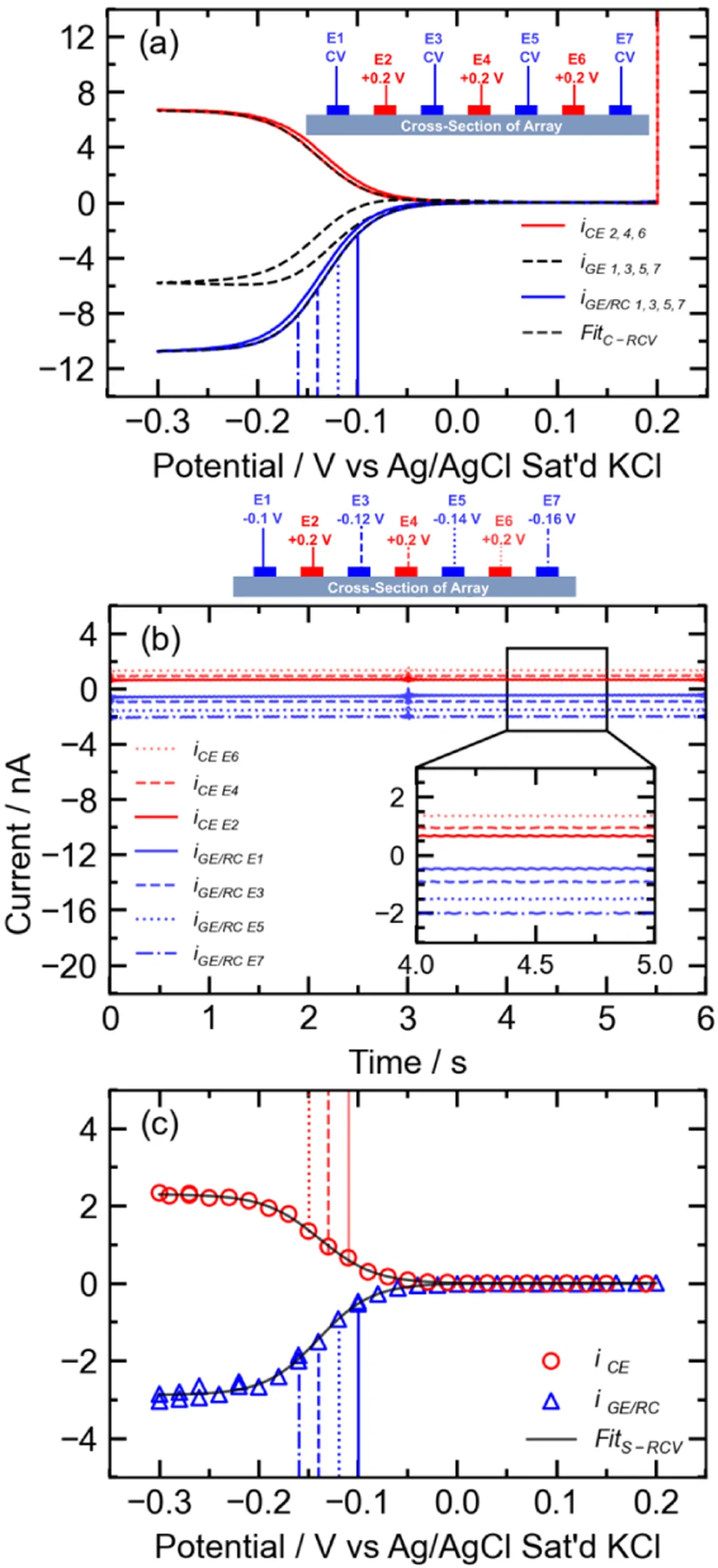
(a) Conventional RCV data obtained at a seven-electrode array, and the transformation of (b) CA data acquired at the same array to produce (c) a final Snapshot RCV response. The solution consisted of a model redox species of 0.50 mM Ru­(NH_3_)_6_Cl_3_ in 0.10 M KCl. In (a), the current is measured at the shorted GEs as they are swept at 0.020 V·s^–1^ from +0.200 V to −0.300 V and back to +0.200 V while the shorted CEs are at open circuit (black dashed curve in the absence of redox cycling), and when the shorted CEs were held at +0.200 V (blue curve, in the presence of redox cycling). The CE current during RCV (red curve) is plotted as a function of the GE potential. The cross section schematic of the array in (a) illustrates the distribution of the potentials during RCV. In (b), individual currents monitored during CA at each of the seven electrodes in the array when the potentials listed in set 7^b^ of Table 1 are applied. The cross section schematic of the array above (b) illustrates the distribution of the potentials for set 7^b^. The relative currents at each GE can be estimated from (a), where the vertical blue lines indicate the potentials applied at different GEs and the red vertical line is the potential, +0.200 V, applied at all the CEs. The inset in (b) is a magnified view of CA responses from 4 to 5 s. In (c), the final Snapshot RCV reconstruction shows the currents for nine sets of applied potentials using the seven-electrode array. Each marker in (c) represents the current average from 4 to 5 s (*N* = 500) for a single electrode. Vertical blue and red lines in (c) point to the GE and CE currents obtained from the CA responses in (b). Each average GE current (blue triangle) is plotted as a function of the potential applied to that electrode; each CE current (red circle) is plotted as a function of the potential halfway between the two adjacent GE potentials. Fits (forward sweep of Conventional RCV, *Fit_C‑RCV_*, dashed black curves (a); Snapshot RCV, *Fit_S‑RCV_*, black solid curves (c)) to the GE and CE responses are also shown.

To further evaluate the Conventional RCV response for the model compound, we compare the magnitude of the steady state currents to predictions made by equations, eq 1 and eq 2. An extensive discussion of these equations and their assumptions and limitations in predicting redox cycling currents at real microband electrode arrays was published previously.^2^ The redox cycling current (|*i*_*lim*_|) predicted by eq 1 is 7.89 nA, where *m* = 3.5 generator-collector pairs (to account for a total of seven electrodes), *w* = 4.08 ± 0.24 μm, and *w*_*gap*_ = 4.14 ± 0.29 μm, and *D*_*avg*_ = 7.45 × 10^–6^ cm^2^·s^–1^ for [Ru­(NH_3_)_6_]^3+/2+^.^51,52^ The current from eq 1 is smaller than |*i*_*GE/RC*_| by a factor of 1.35× and larger than |*i*_*CE*_| by a factor of 1.19×. This is consistent with the trend we observed previously^2^ with similar electrode array dimensions, where the predicted current for the Fe­(CN)_6_^3–/4–^ redox couple fell below the magnitude of the currents at the generator and above that at the collector for a nine-electrode Conventional RCV experiment. Equation 2 additionally accounts for the effect of electroactive sidewalls of the band electrodes, predicts a redox cycling current (|*i*_*lim*_|) of 8.51 nA (where *h* ∼ 0.110 ± 0.005 μm). The magnitude of the current observed at the GEs is 1.25× larger than the predicted current, while for the CEs, the predicted current is 1.28× smaller.

The current trends, observed here and in the previous work, are primarily a consequence of collection efficiency. Equations 1 and 2 assume an ideal collection efficiency of 100%, whereas our experimentally obtained collection efficiency is 62.1% for [Ru­(NH_3_)_6_]^3+^. While feedback from CEs increases the generator current, some of the generated species are lost to the bulk solution, which can be mitigated by increasing the number of electrodes to capture more species. Similar collection efficiencies and amplification factors (as discussed above) have been reported for other microband electrode arrays.^1,2,60^ There is the possibility that a slightly diminished current could be a consequence of residual organics remaining on the electrode surfaces even after plasma cleaning. However, we suspect the presence of insulating residues to be minimal.

### 3.2 Snapshot RCV with a Model Compound

Figure 2(b) shows the CA data acquired for the same array with the same model compound solution when the potentials from Set 7^b^ were individually applied at each electrode and their currents simultaneously monitored for 6 s. The complete CA data acquired for all sets in Table 1 for 0.50 mM [Ru­(NH_3_)_6_]^3+^ can be found in Figure S4 of the Supporting Information. Several features of the raw data confirm the stability in the measurement. First, with a 1 s quiet time, where the electrodes are biased to their respective potentials listed in Table 1, but no current is recorded, the faradaic current transient that is normally expected at the beginning of a CA response is not observed in either Figure 2b or any of the sets of Figure S4. While charging current should be superimposed onto the initial faradaic current, it falls off within milliseconds and is also not observable in the CA data. A minor transient artifact observed at 3 s (due to potential switching at E1) resolves rapidly and does not impact the steady-state generation-collection measured between 4 and 5 s, as seen in the magnified box of Figure 2b. CA responses for GEs, regardless of the set of applied potentials, exhibit an increase in current magnitude, while CA responses for CEs, which are individually biased to +0.200 V, exhibit an increased current magnitude from flux generated by the two neighboring GEs across all sets.

The average CA current response between 4 and 5 s (*N* = 500) for all potential sets in Table 1 where GEs are incremented by 0.020 V per set (Set 2^b^–10^b^) are combined in Figure 2c. The GE responses exhibit an increased current magnitude at potentials more cathodic of *E*_*1/2*_. A sigmoidal shape to the GE responses is observed that mirrors the steady-state response of an interdigitated array under RC conditions. Averaged CA current responses for the CEs are treated similarly and plotted as a function of the average potential of the two neighboring GEs due to their contributions to the incoming flux of [Ru­(NH_3_)_6_]^2+^. At GE potentials more cathodic than *E*_*1/2*_, the CEs exhibit a lower current magnitude than the GEs due to the partial loss of reduced [Ru­(NH_3_)_6_]^3+^ during diffusional transit between electrodes.

The sigmoidal shape of the Snapshot RCV responses is also similar in shape to the current responses of Conventional RCV. To investigate these similarities and initially diagnose deviations from ideal Nernstian behavior, the GE and CE responses of Snapshot RCV and those of the forward sweeps of Conventional RCV were fit to a semiempirical sigmoidal function, eq 3,

$$f\left(x\right)=\frac{\left(A1-A2\right)}{\left(1+e^{\left((x-\theta )p\right)}\right)}+A2$$ 3

where *A1* and *A2* are the current responses at the negative and positive potential limits, respectively, *θ* is the inflection point (half-wave potential, *E*_*1/2*_), and *p* is a parameter defining the steepness of the slope. Parameters for the sigmoidal fits can be found in Table S1 for the Conventional RCV and in Table S2 for the Snapshot RCV in the Supporting Information. The curves for the sigmoidal regression analyses are overlaid with the experimental data for Conventional RCV and Snapshot RCV throughout the figures. The *p* values are related to the heterogeneous electron transfer rate constants. For Conventional RCV, they are 40.5 V^–1^ and 38.2 V^–1^ for GEs and CEs, respectively for experiments involving [Ru­(NH_3_)_6_]^3+^. For Snapshot RCV, the *p* values are 38.5 V^–1^ and 35.8 V^–1^, respectively. As established by Mirkin and Bard^61^ for an uncomplicated electron transfer reaction at a uniformly accessible electrode and in the extreme case when kinetics, *k* ^*0*^, are sufficiently large, *p* can be approximated by $\frac{nF}{RT}$ , which is consistent with fast electron transfer kinetics of [Ru­(NH_3_)_6_]^3+^. Deviations from a purely Nernstian limit ( $\frac{nF}{RT}\approx 38.9{\;V}^{-1}$ at 298 K, *n* = 1) can provide direct evidence of quasi-reversibility.

Figure 3a compares the Conventional RCV response of Figure 2a with the Snapshot RCV responses of Figure 2c. As expected, the Conventional RCV responses exhibit significantly higher currents than the Snapshot RCV responses due to the differing modalities of current monitoring. For Conventional RCV, the current response of the shorted GEs and shorted CEs are summations of currents across the entire array, while for Snapshot RCV, the observed responses are recorded at individual electrodes. To quantitatively compare the magnitudes of the responses, we use the currents at the switching potential obtained from the sigmoidal fits to the data. The ratio of this value from the GE response of the Conventional RCV to that of the Snapshot RCV is 3.69 and the ratio for the CE response between the two techniques is 2.92. These two ratios bracket the average number of electrodes in the array, *m = 3.5*, further confirming that the array’s total current is fundamentally a linear summation of individual steady-state microband responses of the GE-CE pairs. To test this hypothesis, the Conventional RCV data was normalized by *m = 3.5* and overlaid with the individually addressable electrode responses of Snapshot RCV in Figure 3b.

**Figure 3:**
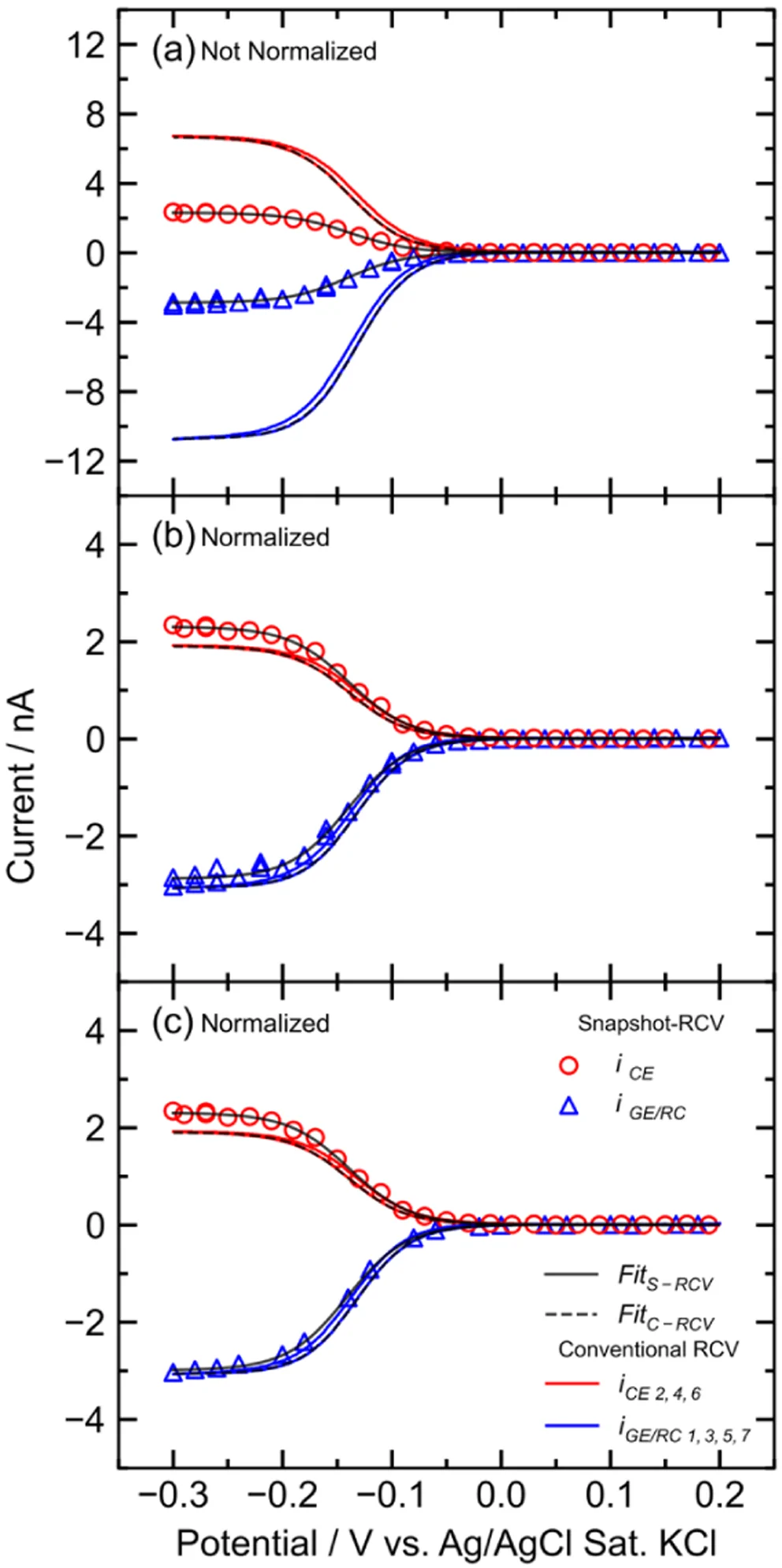
Comparison of Conventional RCV and Snapshot RCV responses with a model redox species of 0.50 mM [Ru­(NH_3_)_6_]^3+^. In (a), Conventional RCV responses for the GEs with redox cycling (blue curve) and CEs (red curve) are overlaid onto Snapshot RCV responses from Figure 2c. In (b), Conventional RCV responses are normalized by generator-collector pairs, *m* = 3.5, and overlaid onto the Snapshot RCV responses from Figure 2c. In (c), the Snapshot RCV responses from Figure 2c are included without the discretized currents of the outer GEs in the array (E1 and E7) due to array edge effects. Sigmoidal fits (forward sweeps of Conventional RCV, *Fit_C‑RCV_*, dashed black curves; Snapshot RCV, *Fit_S‑RCV_*, black solid curves) to the GE and CE responses are also shown.

The reconstructed “spatial” voltammogram shows agreeable overlap with the time-dependent sweep of the conventional method. However, there is a minor negative offset in magnitude of the CE response that suggests an overnormalization of the Conventional RCV response and a minor positive offset in magnitude of the GE response that suggests an under-normalization of the Conventional RCV response. Using a value for *m* that equals half the total number of band electrodes for an array containing only a few electrodes was implemented previously.^2^ Thus, this practice is also applied here. However, we should point out that dividing the GE and CE currents by the actual number of the assigned GE and CE electrodes (four and three, respectively), partially compensates for the small offsets of the normalized Conventional RCV. However, it is difficult to fully justify a true four and three electrode assignment because the flux to the outer GE electrodes and likewise to the CE electrodes adjacent to them, is quite different than between GEs and CEs within the inner region of the array. More discussion about the differing flux across the array is provided below.

Despite the minor scaling variance, the similarity in sigmoidal behavior of Snapshot RCV to that of the Conventional RCV confirms that the new technique captures the steady-state behavior of the system, even when reconstructing the voltammogram spatially rather than temporally. This further suggests that Snapshot RCV can investigate the thermodynamic range in a nonsequential approach, taking “snapshots” of electrochemical behavior across the potential window.

This concept is further demonstrated in Figure 3c, where the outer GEs, E1 and E7, for each set have been excluded from the Snapshot data to remove array edge effects, leaving only the inner, diffusively coupled electrodes. The combined Conventional RCV efficiency is penalized by the uncollected current generated at the outer GEs. The outermost electrodes experience radial diffusion from the semi-infinite bulk solution and redox cycling from only one neighboring collector electrode, lowering the replenishment of recycled analyte in comparison to the interior electrodes. The net effect is a lower generator current than if that electrode had been between two collector electrodes. Because Conventional RCV sums the linear diffusion profile resulting from overlapping diffusion layers during redox cycling at the inner array with this differing flux at the array edges, the global collection efficiency is artificially higher by this disproportionate decrease in GE current. By mathematically excluding these outer currents, Snapshot RCV computationally strips away radial edge effects and isolates the behavior of the inner array, allowing for a postexperimental geometric filtering, something Conventional RCV cannot do. Consequently, the magnitude of the Snapshot GE voltammogram that is fit from the inner array alone as shown in Figure 3c is enhanced in comparison to that for the outer GE current in Figure 3b. A comparison of the fitting parameters for these two cases is provided in Table S2 of the Supporting Information. Because contributing GE current cannot be entirely decoupled from outer CE responses, E2 and E6, we expect a slightly lower contribution of flux arriving from outer GEs at the single neighboring CEs but have not tried to compensate for this effect in our data processing.

The percent collection efficiencies for the Conventional and Snapshot RCV results are provided in Table S3 of the Supporting Information. To obtain these efficiencies, the fit parameters in Tables S2 and S3 of the Supporting Information were used in eq 3 to first calculate the CE and GE currents at the potential at the end of the voltammetric range (e.g., −0.300 V for [Ru­(NH_3_)_6_]^3+^ vs Ag/AgCl (saturated KCl)) and then these values were ratioed. Most noticeable is that the collection efficiency for [Ru­(NH_3_)_6_]^2+/3+^ for Snapshot RCV whose fit includes the outermost GE currents (80.1%) is larger by a factor of 1.29× than that of the Conventional RCV (62.1%). By removing the GE currents from the sigmoidal fit, the collection efficiency for the Snapshot RCV is slightly smaller (79.1%) because the GE current no longer underreports from lower flux at the edges, but its current efficiency is still substantially larger than that for the Conventional RCV.

The relatively lower collection efficiency of Conventional RCV likely arises from a combination of dynamic sweep effects, where the diffusion layer is constantly shifting, and geometric edge effects of the array that are inseparable in the summed response. With CA performed during Snapshot RCV, the diffusion layer can fully develop under a constant potential and stabilization in the steady-state generation-collection can be more efficiently achieved.

### 3.3 Snapshot RCV Applied to Small Biochemical Analytes

We extend the comparison of Conventional and Snapshot RCV methods to a series of biochemical species: 0.50 mM DA, L-DOPA, DOPAC, and AA in aCSF (Figure 4). These species challenge the techniques with a wider range of diffusion coefficients, chemical mechanisms, and electron transfer kinetics. The same seven-electrode subset of the array was used as for [Ru­(NH_3_)_6_]^3+^ but where the Conventional RCV involved sweeping the shorted GE potential at 0.020 V s^–1^ from −0.100 V to +0.550 V to oxidize the analyte while shorted collectors were held at a constant potential of −0.100 V to reduce electroactive generated product back to its starting form. These compounds undergo a two-electron oxidation involving one and two protons at an electrode under potentiodynamic control.^7,8^ Oxidation of the catechol moiety in DA, L-DOPA and DOPAC produces the orthoquinone product and subsequent following chemistries take place. The overall oxidation of AA is to the dehydro-L-ascorbic acid product, also with following chemistry. Conventional RCV responses and the responses normalized to *m* = 3.5 are provided in Figure 4 for each of the compounds. The raw CA responses for GE potential sets 11 through 22 as listed in Table 1 for DA, L-DOPA, DOPAC and AA can be found in the Supporting Information in Figures S5–S8, respectively. The Snapshot RCV results, reconstructed from the CA responses over the interval of 4–5 s, are overlaid with the Conventional RCV in Figure 4 and Figure S9 of the Supporting Information. In Figure 4, CA currents from GEs on the outer edges of the array have been excluded from the Snapshot responses, and in Figure S9, CA currents from GEs on the edges of the array have been retained. Each analyte exhibits its own unique redox cycling voltammogram, with a shape due to electron transfer kinetics as reflected in the p values from the sigmoidal curve fitting, different current magnitudes, and distinctive collection efficiencies.

**Figure 4:**
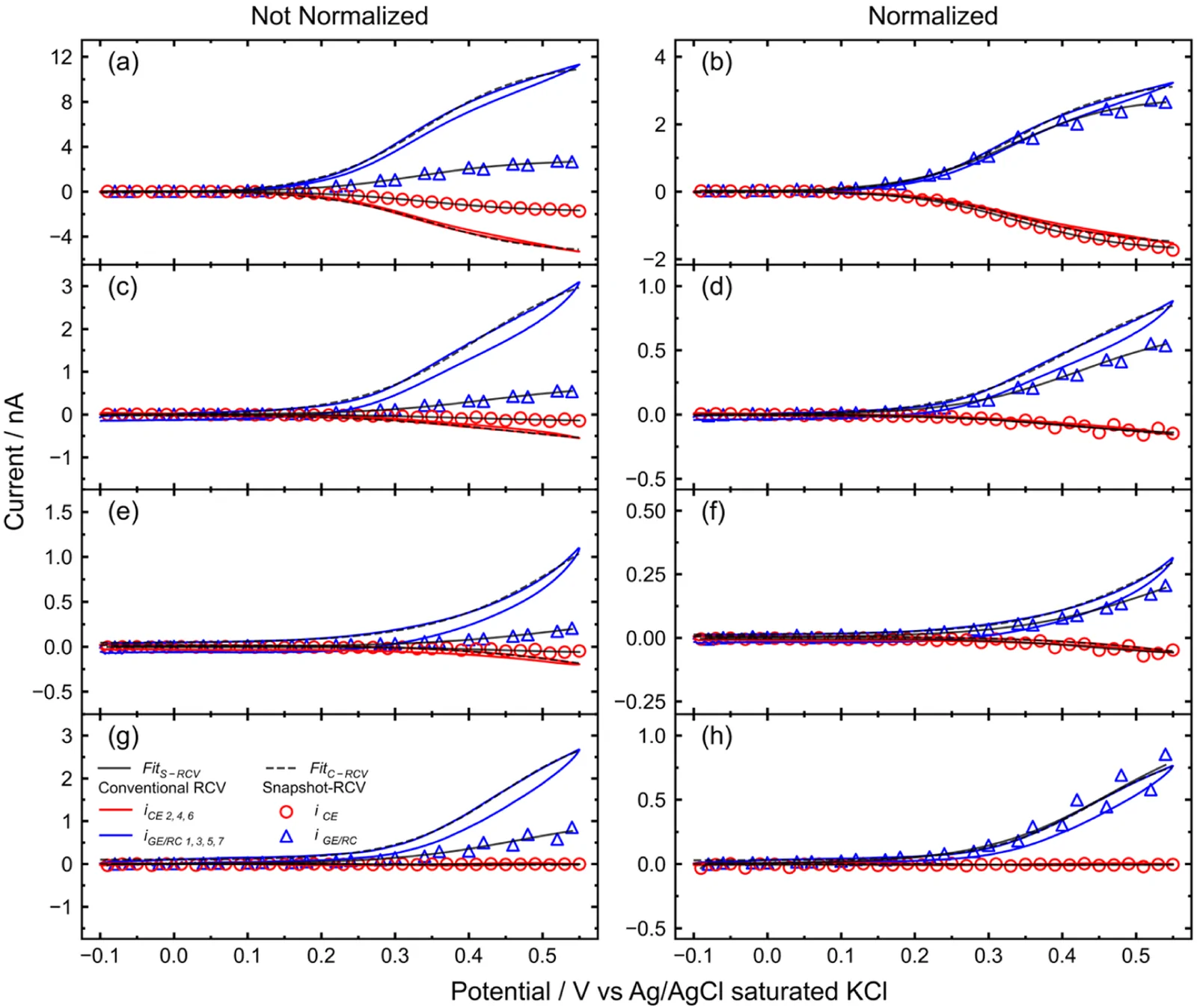
Overlay of Conventional RCV and Snapshot RCV responses for 0.50 mM DA (a–b), 0.50 mM L-DOPA (c–d), 0.50 mM DOPAC (e–f), and 0.50 mM AA (g–h) in aCSF solution. Conventional RCV responses for the GEs (blue curve) and CEs (red curves) are plotted without normalizing by the number of generator-collector electrode pairs, *m* = 3.5, in (a), (c), (e), and (g), and with normalization in (b), (d), (f), and (h). Snapshot RCV responses are overlaid with the Conventional RCV responses for each species and do not include the outer GEs. Sigmoidal fits (forward sweeps of Conventional RCV, *Fit_C‑RCV_*, dashed black curves; Snapshot RCV, *Fit_S‑RCV_*, black solid curves) to the GE and CE responses are also shown.

Conventional RCV for DA is shown in Figure 4a. The observed GE response for DA is more drawn out than that for [Ru­(NH_3_)_6_]^3+^, and the shape is consistent with our previously published work for DA on the same array design.^1,2^ The p values from the sigmoidal fits which quantify the shape, are 14.07 ± 0.08 V^–1^ and 14.04 ± 0.08 V^–1^ for the GE and CE responses, respectively. These values are substantially smaller than the expected Nernstian limit of 77.89 V^–1^ (for *n* = 2), and consistent with relatively slow heterogeneous electron transfer kinetics.^5^

Equations 1 and 2 predict steady state currents of 12.3 nA and 13.3 nA, respectively, using *D*_*DA*_ = 6.0 × 10^–6^ cm^2^·s^–1^,^13^ *n* = 2, and *m* = 3.5. The sigmoidal fits to the experimental data for Conventional RCV yield a GE anodic current for the catecholamine at the switching potential of *i*_*GE/RC,E1,3,5,7*_ = 11.47 nA and a corresponding CE cathodic current for the *o*-quinone form of *i*_*CE,E2,4,6*_ = 5.4 nA. The predicted currents from eqs 1 and 2 are much higher not only because they assume an ideal 100% collection efficiency as was discussed above for [Ru­(NH_3_)_6_]^3+^, but also because of the more complex DA electrochemistry. Additional following chemistry is observable in a measured collection efficiency of only 47.2% for DA that is substantially lower than the 62.1% measured by Conventional RCV for [Ru­(NH_3_)_6_]^3+^ (see Table S3 of the Supporting Information). Dopamine and the other catecholamines follow an ECC’ mechanism. The electron transfer, E, generates an oxidized product of DA, *o*-quinone, which undergoes intramolecular cyclization, C (following chemistry), to form leucoaminochrome, an electrochemically silent product under the conditions used in these experiments. In additional following chemistry, C′, the leucoaminochrome can react with another *o*-quinone, to form the electrochemically silent aminochrome and the original catecholamine, regenerating one DA for every two o-quinones that react.^7,8,46,47^ These reactions diminish the concentrations of both the *o*-quinone species arriving at the collector and the DA returning to the generator. (Surface fouling is not a significant contributor to the relatively low redox cycling currents when compared to the ideal case as was described above and by comparing electrochemical responses before and after the studies in Figure S3 of the Supporting Information.) The collection efficiency for DA by Conventional RCV here is smaller than that reported by us previously (78–83%) for the same array design.^1,2^ However, the previous work used an electrode configuration that favored collection, having more CEs than GEs, and fresh DA from radial diffusion to the narrower five-electrode array is also a contributing factor, which will be discussed further below.

Figure 4a–b shows the “spatial” reconstruction of Snapshot RCV data for 0.50 mM DA when the potentials from Table 1 (sets 12^b^–22^b^) were individually applied to each electrode. The CA responses for each electrode are provided in Figure S5 of the Supporting Information. There is a small current transient that is especially noticeable across the first 3 s in sets 21 and 22, where the GEs are held at the most anodic potentials. Data handling was performed in the same manner as in the [Ru­(NH_3_)_6_]^3+^ studies, and where the current is collected beyond this region (at 4–5 s).

The overlay of the normalized Conventional RCV (by *m* = 3.5) and the Snapshot RCV in Figure 4b aids to visually compare their shapes. The shape of the Snapshot RCV appears to accurately capture that of the Conventional RCV response, in which is embedded the heterogeneous electron transfer kinetics for DA. The p values of 14 ± 1 and 13.5 ± 0.5 from the sigmoidal regression analysis for GE and CE responses, respectively, of the Snapshot RCV, which are within one standard deviation of the values for Conventional RCV, further confirm the similarity (see Tables S1 and S2 of the Supporting Information). (These *p*-values arise from data that exclude currents from GE1 and GE7, which provide a better fit than including them (*R* ^2^ = 0.9947 vs *R* ^2^ = 0.9867, respectively). The unique behavior of the GEs at the array edges are described in more detail below.

The collection efficiency for DA measured by Snapshot RCV (60.0–62.2%) is substantially higher than that by Conventional RCV (47.2%), and by a factor of 1.3×, just as it is for the case of [Ru­(NH_3_)_6_]^3+^. Consistent with Conventional RCV, however, the Snapshot RCV collection efficiency for DA is lower than for [Ru­(NH_3_)_6_]^3+^ due to the complex following chemistry.

Analysis of the GE currents for the Snapshot RCV of DA reveals an interesting trend that is the opposite of that for [Ru­(NH_3_)_6_]^3+^. There is an increased current magnitude at the outer GEs, E1 and E7, (Figure S9a–b of the Supporting Information) and a lower current at the interior GEs (Figure 4a–b). This trend for DA also leads to a lower collection efficiency when the outer GE currents are included in the sigmoidal fits (60.0% vs 62.2%, Table S3 of the Supporting Information). The higher current at the outer GEs for DA arises from the same fundamental concept as for the opposite effect for the [Ru­(NH_3_)_6_]^3+^: the overlapping diffusion layers dominate at the interior electrodes and radial flux dominates at the outer electrodes of the array. In the case of DA, although the interior electrodes benefit from high concentration gradients due to redox cycling, the net linear diffusion from shielding over the array experiences a net loss of DA over time because of the following chemistry, while the edge GEs receive a continued supply of fresh DA by radial flux. This effect is magnified when E7, the outermost GE, is the most anodically poised. There may also be a carry-over of depleted DA from one set of potentials to the next especially on the right side of the array (GE5 and GE7) which were always poised more anodically throughout sequential sets than on the left (GE1 and GE3). This might explain the slightly higher current at the interior GE3 than at the GE5. This depletion effect is also visible within individual CA responses in sets 21 and 22, where the currents gradually decline with time. Each CE current consistently mirrors the currents of its adjacent GEs. When the neighboring GE currents are higher, then the current of the CE between them is understandably higher, and vice versa. This mirroring effect is more easily observed in the cases of L-DOPA, DOPAC, and AA in Figure 4d, f and g, when there are even more variations in current among nearby GEs.

Conventional RCV for 0.50 mM L-DOPA and DOPAC in aCSF was performed in the same way as for DA and the corresponding responses are shown in Figure 4c and e. The shapes are even more drawn out than for DA, with p-values for GE currents of 12.3 ± 0.1 and 10.6 ± 0.1, respectively, and for CE currents of 10.0 ± 0.1 and 10.4 ± 0.1, respectively (Table S1 of the Supporting Information). Collection efficiencies are quite low, at 18% (Table S3 of the Supporting Information). These different behaviors reflect slower heterogeneous electron transfer kinetics and faster following chemistry, compared to DA.

At slow scan rates or low concentrations, such as those used in this work, intramolecular reactions for DOPAC dominate over dimerization reactions,^11^ both of which would diminish concentrations of arriving species at the collector. The shape of the GE response of the Conventional RCV for DOPAC is similar to the literature,^11,62,63^ although at a more anodic potential. It is not clear at this time why the response from DOPAC is much more drawn and pushed to more anodic potentials than expected. The onset of DOPAC oxidation has been shown to increase in acidic solutions,^63^ but is not likely the reason, because our aCSF solution is buffered to pH 7.4. However, the *E*_*1/2*_ for DOPAC has been reported at ∼200 mV greater than DA on a carbon fiber disk electrode in citrate-phosphate buffer at a pH of 7.4.

Figure 4c and e show the Snapshot RCV responses for L-DOPA and DOPAC superimposed on the Conventional RCV results and Figure 4d and f provide overlays with the Conventional RCV data normalized to *m* = 3.5. The sigmoidal function best models the Snapshot data that exclude GE1 and GE7 of the array edges (with *R* ^2^ values of 0.9919 for L-DOPA and 0.9941 for DOPAC, respectively). The Snapshot shapes are similar to the Conventional ones, with p-values for GE currents of 12 ± 1 and 10 ± 1, respectively, and for CE currents of 12 ± 3 and 14 ± 5, respectively (Table S2 of the Supporting Information). There is much more uncertainty in these values than for DA because of larger variation between individual electrode responses and lower currents. Collection efficiencies for Snapshot RCV are low, at 20.0% for L-DOPA and 28.3% for DOPAC, only 0.32× and 0.45× the collection efficiency for DA but are still higher than those obtained from Conventional RCV (Table S3 of the Supporting Information).

Consistent with Snapshot RCV for DA, there is an increased current magnitude at the outer GEs, E1 and E7, (Figure S9a–b of the Supporting Information) and a lower current at the interior GEs (Figure 4c–f) for both L-DOPA and DOPAC. We attribute this behavior to the same reasons as described for DA. Except in these cases, there is a greater variation at the GEs at the array edges than the interior ones because there is even less collection efficiency of the generated product, leading to greater depletion of the analyte during the course of the experiment.

Conventional RCV for 0.50 mM AA in aCSF is shown in Figure 4g. The GE response is drawn out due to slow heterogeneous electron transfer kinetics, with a p value of 14.48 ± 0.08 (Table S1 in the Supporting Information), and has a shape similar to previously reported results at single electrodes.^62^ Redox cycling is not expected to occur as the oxidation of AA generates dehydroascorbic acid, an electrochemically silent product subject to rapid dehydration.^9^ Thus, collection efficiency is zero because there is no measurable faradaic current at the CEs. These circumstances and the zero current also provide evidence that the array signal is free from interelectrode capacitive crosstalk.

Figure 4g shows the Snapshot RCV response for AA superimposed on the Conventional RCV results and Figure 4h provides an overlay that matches well with the Conventional RCV data normalized to *m* = 3.5. The sigmoidal function is a poor fit for Snapshot data that both include (*R* ^2^ = 0.9296) and exclude (*R* ^2^ = 0.9587) currents from GE1 and GE7 at the edges of the array. The Snapshot shape for the GE current is similar to and has a p-value (12 ± 3) within error of the Conventional shape (Table S2 of the Supporting Information). As in the Conventional RCV case, there is no measurable faradaic current at the CEs. At this time, we are unable to explain the trend of the GE currents for AA across the array for a given set of potentials, where the GEs at the edges have lower current than the interior GEs. This is the reverse of the trend for DA, L-DOPA, and DOPAC, and more similar to that for [Ru­(NH_3_)_6_]^3+^. However, electronic noise may play a larger role in the AA signal, because of the low currents due to a lack of RC amplification.

Given the results for DA, L-DOPA, DOPAC and AA, that exhibit following chemistry, a true steady state might not be achievable for chemistry that undergoes fast following chemistry and when the breadth of the array is large enough and gaps between electrodes wide enough to promote a net depletion of analyte. Smaller gaps between GEs and CEs would lead to more efficient reversal of products before loss to following chemistry. Narrower arrays that allow radial diffusion of fresh analyte could offset analyte depletion from chemical reactions. Faster data acquisition and fewer potential sets would minimize chemical conversion. Design of arrays and experiments for use with Snapshot RCV to perform chemical sensing should consider how to balance fast data acquisition, time scales for recycling across gaps between GEs and CEs, evolution of diffusion profiles across the breadth of the array, relative contributions from radial flux and overlapping diffusion layers, and redundancy to achieve amplification.

## 4 Conclusions

Snapshot RCV combines the potential-resolved information of Conventional RCV with the temporal sampling capabilities of chronoamperometry. In principle, the current at a complete set of electrode potentials is recorded as soon as the instrumentation responds, double-layer charging has diminished, and redox molecules have bidirectionally diffused across the interelectrode gap. This point may be reached in less than 1 s. The individual generator and collector currents are plotted across the potential range, forming “spatial” voltammograms. The positions and shapes of the resulting wave forms, together with their collection efficiency, provide information about the thermodynamics, heterogeneous electron transfer kinetics, following chemistry and mass transport of the redox species. These features of the electrochemical response serve as a fingerprint to identify the analyte, and the current magnitude indicates the analyte amount. Snapshot RCV was demonstrated using a model system ([Ru­(NH_3_)_6_]^3+^), and then applied to DA, L-DOPA, DOPAC, and AA which have more complex chemical and kinetic properties.

The potentials applied to the GEs and CEs across the array can be selected to cover the thermodynamic range of interest, and the resolution is limited by the number of independently addressable electrodes and instrument channels. If greater resolution is desired, different sets of potentials can be applied in sequence, as was demonstrated here, with a total experimental time equal to the number of sets multiplied by their acquisition periods. Pausing between sets might be needed for analyte concentrations to recover; microfluidics delivery of fresh solution could eliminate the need for this delay.

An interesting feature of Snapshot RCV is that it distinguishes currents at the outer GEs and CEs from those at inner electrodes. This is important because the outer electrodes experience different mass transport than the inner electrodes and can either increase or decrease the total current, depending on the analyte. In Conventional RCV these contributions are included in the summed response and can alter the measured collection efficiency.

Snapshot RCV trades off signal amplification for thermodynamic resolution. Conventional RCV, in contrast, trades off speed for amplification. One way to address the tradeoff in Snapshot RCV is to add more electrodes, increasing the electroactive footprint, and repeating the electrode-potential pattern across the array. For example, grouping 28 individually addressable electrodes into four subset arrays and shorting every seventh electrode together could provide a 4-fold amplification. It may be possible to achieve a 4-fold amplification within the original electroactive footprint by decreasing the electrode widths and interelectrode gaps, as shown previously for Conventional RCV using computer simulations.^2^ Effects on the Snapshot RCV response due to proximity and geometry of physically grouped subsets of arrays would need further investigation.

The ability to monitor Snapshot RCV responses at a fixed potential distribution over time can capture transient events, including those in which the potential of the response shifts as conditions change. The formal potential of proton-coupled redox species such as hydroquinones and catechols shifts with pH,^64,65^ as does the pH-dependent reduction window of dissolved oxygen sensing, which can overlap or move relative to other analytes.^42,66^ The reference is another source of drift, because quasi- and pseudoreference electrodes respond to changes in solution composition.^67^ Temperature contributes as well, shifting the formal potential of the redox couple and the reference together.^68^ Fixed potential dual-electrode chronoamperometry detects the resulting current changes, but not where the response along the potential axis has moved. Snapshot RCV records the potential-resolved response over time, locating such shifts and distinguishing them from changes in the current magnitude.

Snapshot RCV may also track transient events in which the response itself evolves as the chemistry changes in time, such as the loss in collection efficiency as electrogenerated species degrade or electrodes foul.^45^ or the appearance of new features as products and intermediates accumulate during electrosynthesis and related chemical reactions that have been conventionally monitored in the past by single electrode CV and CA.^53^

Snapshot RCV has features that would make it suitable as a detector for chromatography and electrophoresis. Generation-collection detection has already been implemented across a wide range of electrode scales for separations, from macroscale dual-electrode detectors and IDA redox cycling^59,69^ at fixed potentials in liquid chromatography to coplanar dual-electrode detectors integrated with microchip electrophoresis.^58^ It has been demonstrated that applying different oxidizing potentials at individual electrodes in an array can disambiguate coeluting or comigrating species^57^ that a single detection potential cannot resolve in real samples.^55^ Snapshot RCV unifies these approaches by delivering an identifying voltammetric profile rapidly enough to characterize a solute as it elutes or migrates, adding identification to the quantification that fixed-potential redox-cycling detectors provide. In conclusion, the combination of speed, full voltammetric profile, signals for both generation and collection, and scalable electrode configuration that preserves a small interelectrode gap allows Snapshot RCV to occupy a distinct and useful niche.

## Supplementary Material



## Notes

Supporting Information contains discussions on CV characterizations from pre- and postexperimental analysis, calculations of collection efficiency, and fluctuations observed in raw CA data, as well as an image of the microelectrode array device (Figure S1) and microscopic image of array on device (Figure S2); Figure S3: plot of CV responses; Tables S1 and S2: sigmoidal fit parameters for forward sweeps of GE and CE responses of Conventional RCV and GE and CE responses of Snapshot RCV; Table S3: %Collection efficiency determined from fit parameters in Tables S1 and S2; Figures S4–S8: plot of raw CA data for [Ru­(NH_3_)_6_]^3+^, DA, L-DOPA, DOPAC, and AA, respectively; Figure S9: plot of Snapshot RCV data including outer GEs and Conventional RCV overlay for DA, L-DOPA, DOPAC, and AA (PDF)
