# Supplementary material for: Snapshot Redox Cycling Voltammetry (Snapshot RCV): Fast Generation-Collection Detection across a Wide Potential Range at a Chip-Based Microelectrode Array
Source: ACS Meas Sci Au. 2026 Jul 22;6(4):1223–38. doi: 10.1021/acsmeasuresciau.6c00091 (PMC13495736; doi:10.1021/acsmeasuresciau.6c00091)
Supplement: Supplementary file 1 [file tg6c00091_si_001.pdf]

Supporting Information

Snapshot Redox Cycling Voltammetry (Snapshot RCV): Fast Generation-Collection Detection Across a Wide Potential Range at a Chip-Based Microelectrode Array

*Blake A. Sterling, Miguel Á. Ábrego Tello, Ingrid Fritsch**

Department of Chemistry and Biochemistry. University of Arkansas. 345 N Campus Walk. Fayetteville, AR, 72701, USA.

**To whom correspondence should be addressed: ifritsch@uark.edu*

Supporting Information Table of Contents

1. Content of Supporting Information....................4
2. CV Characterization of Individual Electrodes Using a Model Compound. 5
3. Determining Collection Efficiency from Sigmoidal Fit Parameters....................7
4. Comment on Fluctuations in Raw CA Data from Figures S4-S8 8
5. Tables 9
   5.1. Table S1: Fit Parameters for Conventional RCV from Semi-Empirical Sigmoidal Function 10
   5.2. Table S2: Fit Parameters for Snapshot RCV from Semi-Empirical Sigmoidal Function 11
   5.3. Table S3: Percent Collection Efficiencies from Sigmoidal Fits 13
6. Figures....................14
   6.1. Figure S1: Microband Electrode Array Device Image 14
   6.2. Figure S2: Microscopic View of Microband Electrode Array.................... 15
   6.3. Figure S3: Preexperiment and Postexperiment CV Responses in Model Compound Solution.................... 16
   6.4. Figure S4: CA Data for Snapshot RCV Potential Sets from Table 1 Using a Model Compound. 17

6.5. Figure S5: CA data for Snapshot RCV from Potential Sets in Table 1 using 0.50 mM DA in aCSF 19

6.6. Figure S6: CA data for Snapshot RCV from Potential Sets in Table 1 using 0.50 mM L-DOPA in aCSF 21

6.7. Figure S7: CA data for Snapshot RCV from Potential Sets in Table 1 using 0.50 mM DOPAC in aCSF 23

6.8. Figure S8: CA data for Snapshot RCV from Potential Sets in Table 1 using 0.50 mM AA in aCSF 25

6.9. Figure S9. Overlay of GE and CE currents for conventional RCV and snapshot RCV (including the flanking GEs) for DA, L-DOPA, DOPAC and AA in aCSF 27

7. References 29

1. Content of Supporting Information

Supporting Information contains discussions on cyclic voltammetry (CV) characterizations from pre- and postexperimental analysis, calculations of collection efficiency, and fluctuations observed in raw chronoamperometry (CA) data, as well as an image of the microelectrode array device (Figure S1) and microscopic image of array on device (Figure S2); Figure S3: plot of CV responses; Tables S1 and S2: Sigmoidal fit parameters for forward sweeps of generator electrode (GE) and collector electrode (CE) responses of Conventional redox cycling voltammetry (RCV) and GE and CE responses of Snapshot RCV; Table S3: %Collection efficiency determined from fit parameters in Tables S1 and S2; Figures S4-S8: plot of raw CA data for hexaammineruthenium(III) ($[Ru(NH_3)_6]^{3+}$), dopamine (DA), 3,4-dihydroxyphenylalanine (L-DOPA), 3,4-dihydroxyphenylacetic acid (DOPAC), and ascorbic acid (AA), respectively; Figure S9: plot of Snapshot RCV data including outer GEs and Conventional RCV overlay for DA, L-DOPA, DOPAC, and AA.

2. CV Characterization of Individual Electrodes Using a Model Compound.

Individual electrodes of the chip-based microband electrode array devices were characterized in a solution containing 0.50 mM $[Ru(NH_3)_6]Cl_3$ and 0.10 M KCl to assess their electrochemical behavior prior to and after experiments. CV current responses for the seven-electrode subset of the array showing diffusion-limited faradaic current for the one-electron reduction of $[Ru(NH_3)_6]^{3+}$ to $[Ru(NH_3)_6]^{2+}$ are sigmoidal in shape at a slow scan rate of 0.020 V s^{-1}, as seen in Figure S1. The magnitude of the current is relatively flat across the plateau of the forward sweep for each of the seven electrodes (N=7). The average of the current from -0.200 V to -0.300 V vs Ag/AgCl (saturated KCl) in the forward sweep obtained prior to experimentation was 2.40 ± 0.04 nA. and that obtained after experimentation was 2.18 ± 0.09 nA. The percent drop in current between pre- and postexperiment characterizations relative to the pre-experiment current is 9.13 ± 3.18%. The GEs exhibited the largest changes in current responses. We postulate this to be caused by modifications to the electrode surfaces and fouling that are more prevalent during catechol oxidation. Dopamine, for example, is known to undergo polymer formation and auto-oxidation at physiological pH's (pH = 7) in PBS.[1, 2] Unlike the GEs, which are held at anodic potentials during RCV of the catechols, the CEs are consistently poised at sufficiently reducing potentials, and as such, would experience less adverse surface effects. Thus, we expect there to be variations in electrode sensitivity across the array over a series of experiments, with deviations consistent with the changes described above.

To confirm that these microfabricated electrodes, which have undergone substantial processing, were sufficiently cleaned, we compare the magnitude of the diffusion-limited current for the model compound $[Ru(NH_3)_6]^{3+}$ to predictions made by mathematically derived equations. Figure S1 shows that the magnitudes of plateau currents for CV at the individual microband

electrodes are similar to the quasi-steady state current, $|i_{qss}|$, for a potential step experiment using the approximations of eq S1[3-5]and eq S2[6, 7]

$$i(t) = \frac{2\pi nFlDc^*}{\ln\left(\frac{64Dt}{w^2}\right)} \; for \; \frac{Dt}{w^2} \geq \frac{10}{4} \tag{S1}$$

$$i(t) = nFc^*Dl\sum_{K=0}^{k} C_k \left[\frac{1}{\ln\left(\frac{Dt}{w^2}\right)+b}\right]^k \quad \begin{array}{|l} b = \ln(64) - 2\gamma_E \\ C_0 = 0 \\ C_1 = 2\pi \\ C_2 = -2\pi\gamma_E \\ C_3 = 2\pi\left(\gamma_E^2 - \frac{\pi^2}{6}\right) \\ C_k \ldots \end{array} \tag{S2}$$

where l is the length of the electrode; D is the diffusion coefficient for both the oxidized (O) and reduced (R) species; F is the Faraday constant; n is the moles of electrons transferred per mole of electroactive species; c^* is the bulk solution concentration of the redox species; w is the width of the electrode; t is the time; γ_E denotes the Euler Gamma constant; C_k is a series of k nonzero terms, up to $K = 15$. The calculations of $|i_{qss}|$ displayed in Figure S3 from eqs S1 and S2 used the following parameters: $c^* = 5 \times 10^{-7}$ moles/cm^3, $D_{avg} = 7.45 \times 10^{-6}$ cm^2·s^{-1},[8, 9] $l = 97.17 \times 10^{-4}$ cm , w = 4.08×10^{-4} cm, t = 8.75 s. The time was estimated from the sweep time of the CV response at $E_{½}$

(the potential at the inflection point of the sigmoid response, (- 0.126 V)) and the potential at which the plateau current is measured (-0.300 V vs Ag/AgCl (saturated KCl)) for a scan rate of 0.020 V/s.

Our previously published work compares the $|i_{qss}|$ predicted for these (and other) approximations.[10] Equation S1 is often used and is simpler. Equation S2 is arguably the more accurate one. However, the current obtained from eq S1 is only 4% smaller than that from eq S2, as can be seen in Figure S3. The magnitude of the current plateau for the experimental CV responses in Figure S3 fall at or just below the $|i_{qss}|$ predictions. We had previously shown that the enhanced flux of redox species to the electroactive edges (accounting for electrode height) and the ends of these relatively short band electrodes actually contribute to a larger current than predicted by the approximations We did not investigate further the sources for the small differences between the experimental response and the predicted currents from eqs S1 and S2. It is the consistent electrochemical response across the electrodes in the array that is most important in the evaluation of the Snapshot RCV method.

3. Determining Collection Efficiency from Sigmoidal Fit Parameters

Fit parameters from Table S1 and S2 were reinserted into the semi-empirical sigmoidal function to determine theoretical current responses, Table S3 across the potential window for the forward sweeps of GEs and CEs of Conventional RCV and the GE and CE responses of Snapshot RCV. The efficiencies for each analyte were then calculated for Conventional RCV and Snapshot RCV with and without out GEs included from each current calculation across the incremented potential window. Conventional RCV and Snapshot RCV fit parameters from Table S1 and S2 for $[Ru(NH_3)_6]^{3+}$, DA, L-DOPA, and DOPAC were reinserted into the equation and current magnitude calculated for GEs and CEs at the switching potential of -0.300 V for $[Ru(NH_3)_6]^{3+}$ and +0.550 V

for DA, L-DOPA, and DOPAC. Collection efficiency was determined by taking the ratio of CE/GE current at the switching potential and are reported in Table S3. Because a faradaic current was not measurable at CEs for the AA solutions, collection efficiencies were not calculated for AA

4. Comment on Fluctuations in Raw CA Data from Figures S4-S8

Fluctuations early in the current response are observed in Set 1[a] and only partially in Set 2[b] of Figure S4 and Set 11[a] and only partially in Set 12[b] of Figures S5-S8. The exact reason is unknown. However, these fluctuations led us to use a later time of 4-5 s in the CA responses with which we constructed the Snapshot RCV responses.

5. Tables

5.1. Table S1: Fit Parameters for Conventional RCV from Semi-Empirical Sigmoidal Function

Table S1. Fit parameters for Conventional RCV obtained from semi-empirical sigmoidal function. Function included at end of table.[11] c^* = 0.50 mM. In the case of AA, the collector current was zero, and therefore curve fitting was not performed.

	**Conventional RCV GE (Forward Sweep)**					**Conventional RCV CE (Forward Sweep)**				
	***A1* / nA**	***A2* / nA**	***θ* / V**	***p* / V^{-1}**	***R^2***	***A1* / nA**	***A2* / nA**	***θ* / V**	***p* / V^{-1}**	***R^2***
**$[Ru(NH_3)_6]^{3+}$**	-10.7285 *± 0.0026*	0.0368 *± 0.0015*	-0.1316 *± 0.0000*	40.539 *± 0.047*	0.9999	6.6651 *± 0.0024*	0.0290 *± 0.0013*	-0.1394 *± 0.0000*	38.184 *± 0.059*	0.9999
**DA**	-0.1355 *± 0.0095*	11.474 *± 0.027*	0.3393 *± 0.0005*	14.068 *± 0.078*	0.9991	0.0324 *± 0.0042*	-5.439 *± 0.013*	0.3461 *± 0.0005*	14.042 *± 0.075*	0.9992
**L-DOPA**	-0.0147 *± 0.0026*	3.506 *± 0.018*	0.4113 *± 0.0099*	12.350 *± 0.088*	0.9988	-0.0136 *± 0.0007*	-0.7288 *± 0.0086*	0.4493 *± 0.0025*	10.02 *± 0.11*	0.9977
**DOPAC**	0.0424 *± 0.0097*	2.034 *± 0.052*	0.5500 *± 0.0045*	10.56 *± 0.12*	0.9979	0.0186 *± 0.0002*	-0.385 *± 0.012*	0.5500 *± 0.0051*	10.41 *± 0.13*	0.9974
**AA**	0.0991 *± 0.0014*	3.179 *± 0.014*	0.4419 *± 0.0007*	14.482 *± 0.079*	0.9992	NA	NA	NA	NA	NA

$$f(x) = \frac{(A1 - A2)}{(1 + e^{(x-\theta)p})} + A2$$

5.2. Table S2: Fit Parameters for Snapshot RCV from Semi-Empirical Sigmoidal Function

Table S2. Fit parameters for Snapshot RCV obtained from semi-empirical sigmoidal function. Function included at end of table.[11] Parameters are provided for the case when the outer GEs, E1 and E7, are included and for the case when the outer GEs are excluded. c^* = 0.50 mM. In the case of AA, the CE current was zero and therefore curve fitting was not performed.

	**Snapshot RCV GE (E1 and E7 included)**					**Snapshot RCV GE (E1 and E7 excluded)**				
	A1 **/ nA**	***A2*** **/ nA**	θ **/ V**	p **/ V^{-1}**	R^2	***A1*** **/ nA**	***A2*** **/ nA**	θ **/ V**	p **/ V^{-1}**	R^2
**$[Ru(NH_3)_6]^{3+}$**	-2.878 *± 0.042*	0.010 *± 0.027*	-0.1401 *± 0.0022*	37.3 *± 3.0*	0.9969	-2.912 *± 0.042*	0.006 *± 0.027*	-0.1392 *± 0.0022*	38.5 *± 3.0*	0.9971
**DA**	-0.011 *± 0.039*	2.86 *± 0.11*	0.3422 *± 0.0079*	13.2 *± 1.1*	0.9867	0.011 *± 0.036*	2.791 *± 0.087*	0.3312 *± 0.0066*	14.1 *± 1.1*	0.9947
**L-DOPA**	-0.0035 *± 0.0022*	0.73 *± 0.10*	0.390 *± 0.027*	13.4 *± 3.4*	0.9026	-0.0049 *± 0.0081*	0.688 *± 0.063*	0.425 *± 0.018*	11.8 *± 1.4*	0.9919
**DOPAC**	0.0008 *± 0.0094*	0.32 *± 0.13*	0.469 *± 0.075*	11.2 *± 4.0*	0.8519	0.0018 *± 0.0023*	0.391 *± 0.096*	0.54 *± 0.05*	10.2 *± 1.2*	0.9941
**AA**	0.008 *± 0.017*	0.82 *± 0.14*	0.439 *± 0.032*	13.0 *± 2.9*	0.9296	0.0036 *± 0.0234*	1.09 *± 0.33*	0.465 *± 0.058*	11.6 *± 3.2*	0.9587

Table S2 continued.

	Snapshot RCV CE				
	A1 **/ nA**	***A2*** **/ nA**	***θ*** **/ V**	***p*** **/ V^{-1}**	**R^2**
**$[Ru(NH_3)_6]^{3+}$**	2.307 ± *0.013*	-0.0002 ± *0.0084*	-0.1387 ± *0.0009*	35.8 ± *1.0*	0.9992
**DA**	0.0369 ± *0.010*	-1.759 ± *0.028*	0.3398 ± *0.0033*	13.54 ± *0.51*	0.9982
**L-DOPA**	0.0013 ± *0.0056*	-0.164 ± *0.031*	0.398 ± *0.039*	11.6 ± *3.3*	0.9217
**DOPAC**	-0.0037 ± *0.0022*	-0.069 ± *0.019*	0.439 ± *0.049*	14.0 ± *5.1*	0.8688
**AA**	NA	NA	NA	NA	NA

$$f(x) = \frac{(A1 - A2)}{(1 + e^{(x-\theta)p})} + A2$$

5.3. Table S3: Percent Collection Efficiencies from Sigmoidal Fits

Table S3: Percent collection efficiencies expressed as the ratio of the CE and GE currents that are calculated from eq 3 of the main document (which is also listed at the end of Tables S1 and S2) using the fit parameters in Tables S1 and S2 at (a) -0.300 V for $[Ru(NH_3)_6]^{3+}$ and (b) +0.550 V for DA, L-DOPA, and DOPAC. In (c), the case of AA, the CE current was zero.

	$[Ru(NH_3)_6]^{3+}$ [a]	DA[b]	L-DOPA[b]	DOPAC[b]	AA[c]
**Snapshot RCV – With Outer GEs**	80.1	60.0	21.5	25.3	0
**Snapshot RCV – Inner GEs Only**	79.1	62.2	20.0	28.3	0
**Conventional RCV**	62.1	47.2	18.1	17.6	0

6. Figures

6.1. Figure S1: Microband Electrode Array Device Image

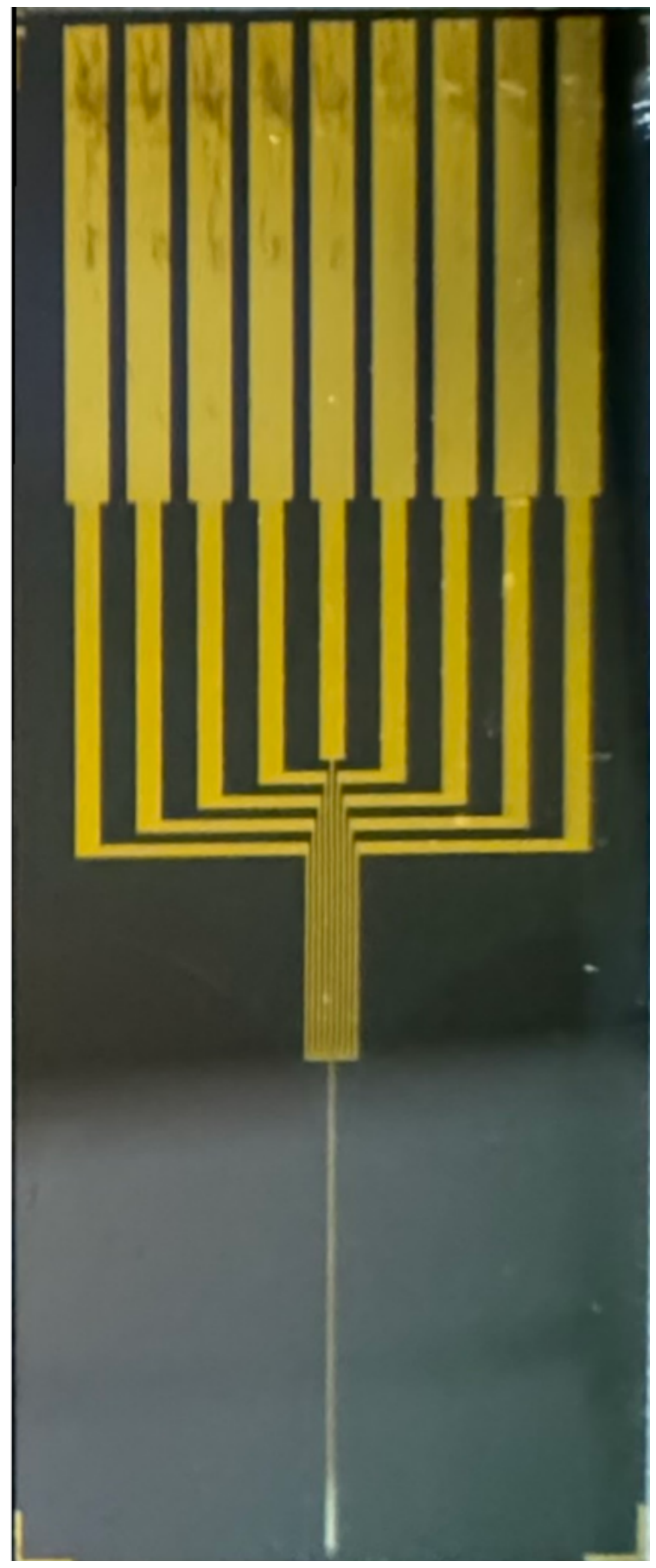

Figure S1. Image of microelectrode array device, showing gold patterned on a chip (27 mm × 11 mm) diced from a silicon wafer insulated with SiO_2. The design was described in our previous work.[12] Devices were partially fabricated at the University of Kansas in the KU Nanofabrication facility. Leads from the contact pads on one side of the chip reach nine individually addressable electrodes on the opposite side, with an electroactive area of 70 µm by 100 µm defined by an opening in an overlying polyimide insulation layer (slightly darker coloring over the leads). A magnified view of the microband electrode array is shown in Figure S2.

6.2. Figure S2: Microscopic View of Microband Electrode Array

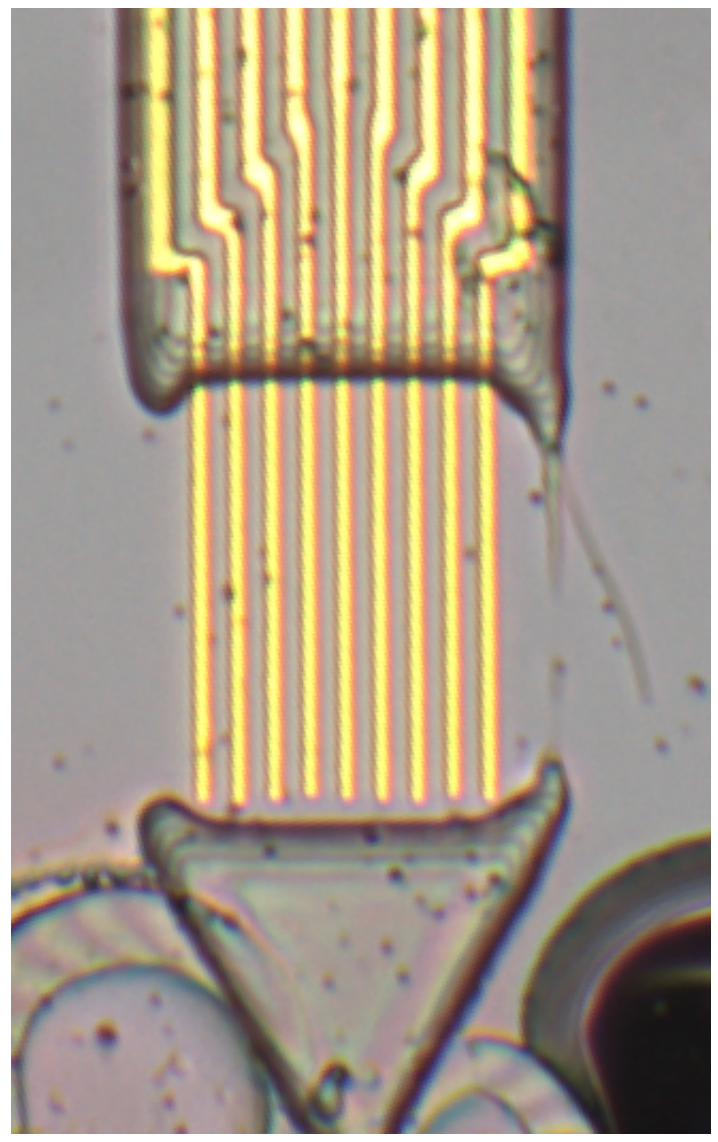

Figure S2. Optical micrograph of a microband electrode array used for the studies herein. The gold electrodes are 97.17 ± 0.94 μm long, *l*, 4.08 ± 0.24 μm wide, *w*, and separated by a 4.14 ± 0.29 μm wide gap, w_{gap}. The raised, transparent film above and below the electrodes in the image is the polyimide insulation layer. The photomask for this layer creates sidewalls that flank the array, outlining a rectangular opening. Their absence in this device, from overdeveloping or under-exposing that insulation layer during fabrication, avoided partial overlap with the outermost electrodes, which sometimes renders them inactive, as was observed in our previously-reported device.[12]

6.3. Figure S3: Preexperiment and Postexperiment CV Responses in Model Compound Solution

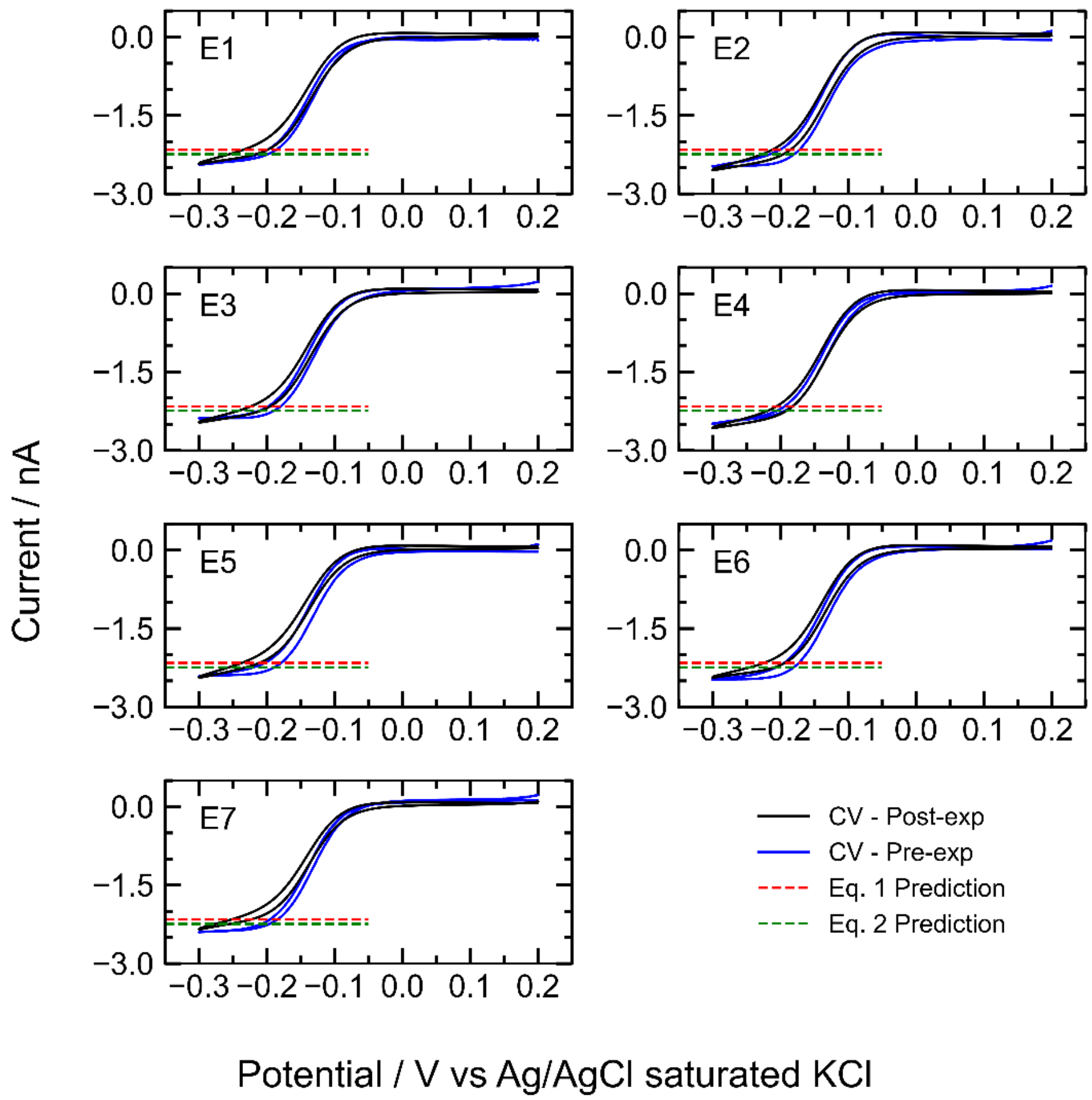

Figure S3. Preexperiment (blue curves) and postexperiment (black curves) CV responses at each microband electrode (E1 through E7) in the array used for all experiments described in the manuscript, performed in a solution containing 0.50 mM $[Ru(NH_3)_6]Cl_3$ and 0.10 M KCl at 0.020 V s^{-1}. The red dashed line indicates the expected faradaic current predicted by eq S1 (2.16 nA), and the green dashed line indicates the expected faradaic current predicted by eq S2 (2.24 nA). The parameters used for the calculations are listed in the text.

6.4. Figure S4: CA Data for Snapshot RCV Potential Sets from Table 1 Using a Model Compound.

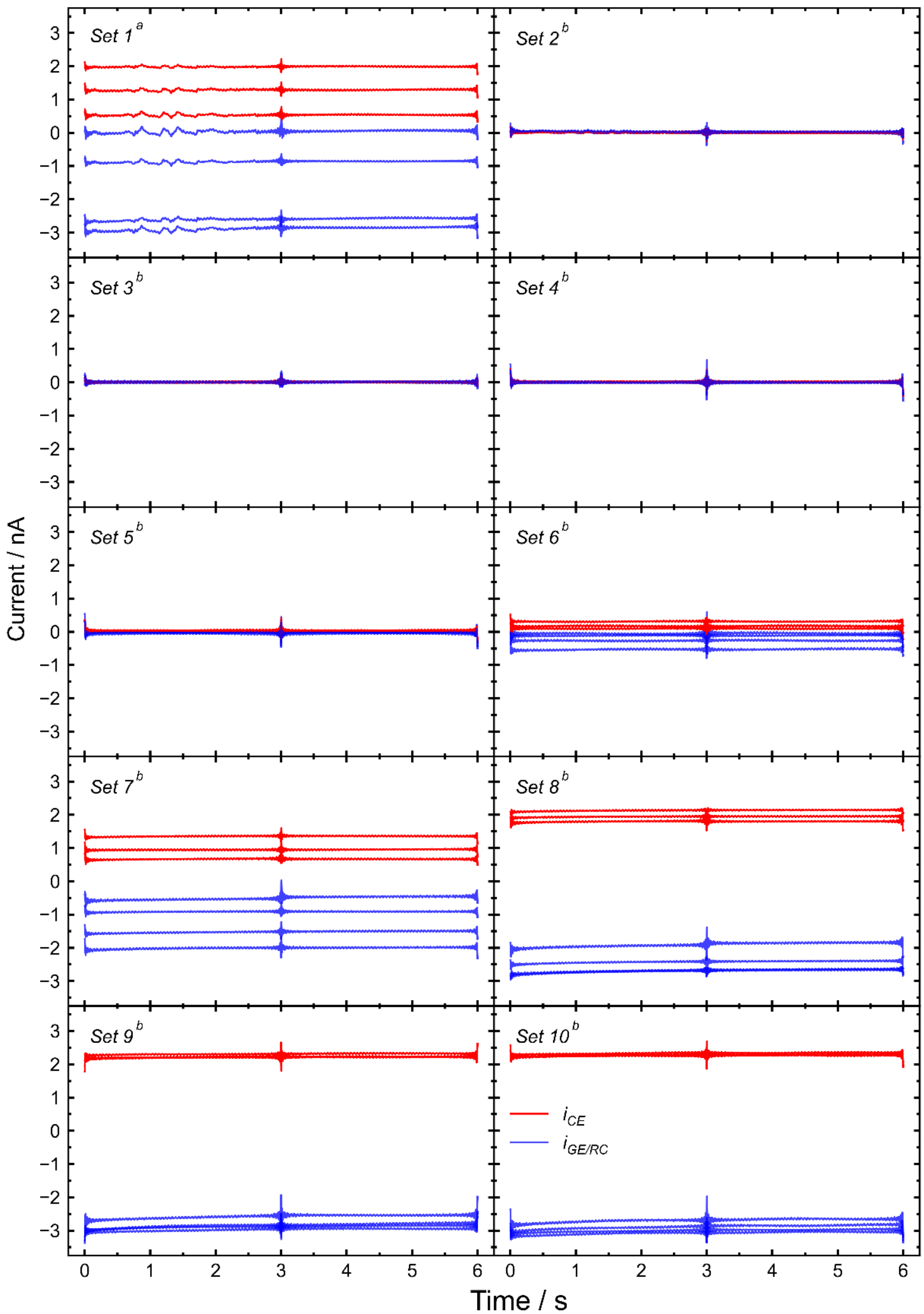

Figure S4. Raw Snapshot RCV CA data for sets 1[a] – 10[b] listed in Table 1 of the main document for a solution of 0.50 mM $[Ru(NH_3)_6]Cl_3$ in 0.10 M KCl. Generator (blue) and collector (red) electrodes are individually biased to the potentials listed in Table 1 and held constant for 6 s with a 1 s quiet time. The GEs of set 1 with an "*a"* superscript are incremented by 0.060 V between electrodes while CEs are held constant at + 0.200 V vs Ag/AgCl (saturated KCl). The GEs with a "*b*" superscript are incremented by 0.020 V per generator per set while the CEs are held constant at + 0.200 V vs Ag/AgCl (saturated KCl).

6.5. Figure S5: CA data for Snapshot RCV from Potential Sets in Table 1 using 0.50 mM DA in aCSF

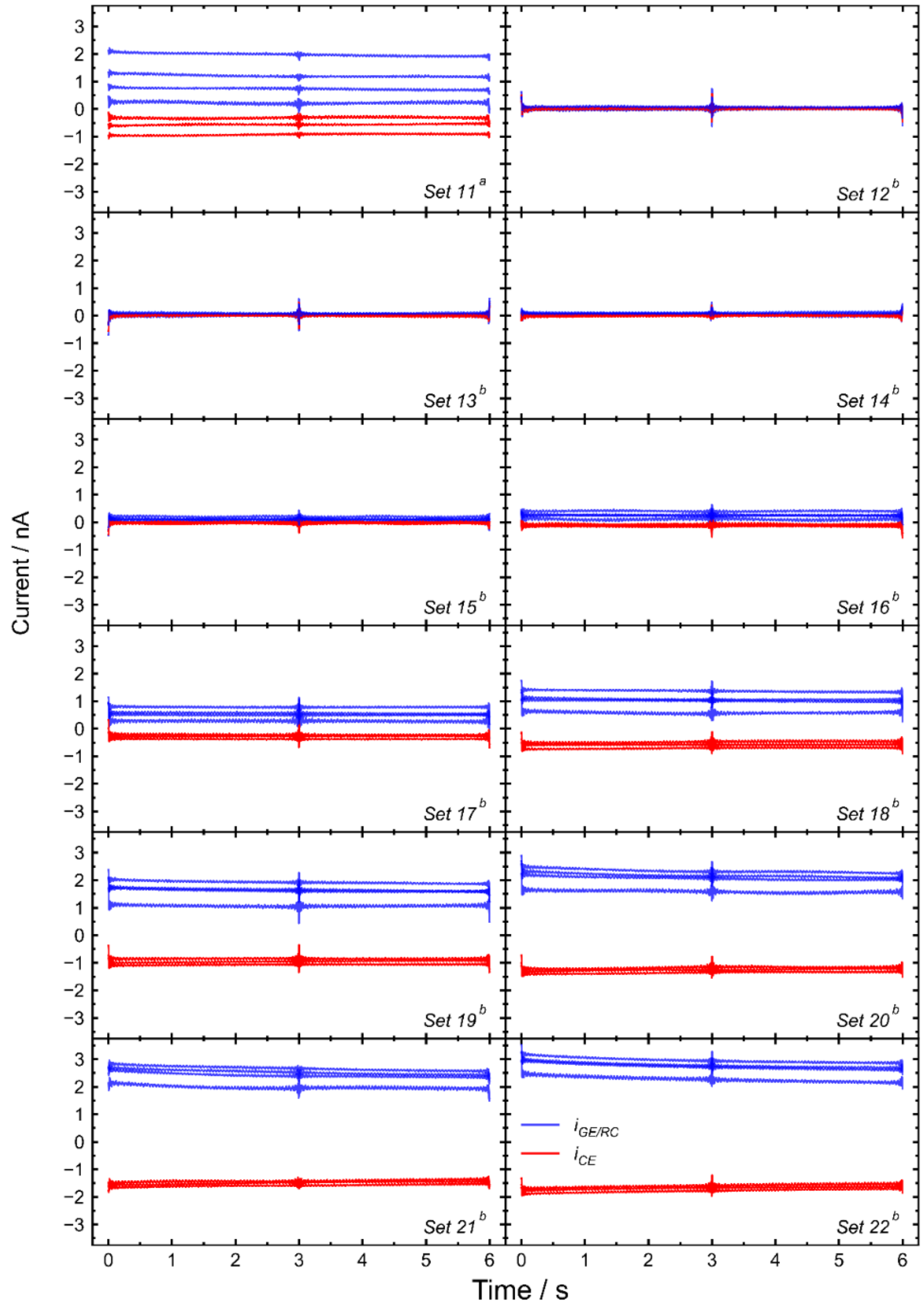

Figure S5. Raw snapshot RCV CA data for sets 11[a] – 22[b] listed in Table 1 of the main document for a solution of 0.50 mM dopamine (DA) in aCSF. Generator (blue) and collector (red) electrodes are individually biased to the potentials listed in Table 1 and held constant for 6 s with a 1 s quiet

time. The GEs of set 11 with an "*a"* superscript are incremented by 0.060 V between electrodes while CEs are held constant at – 0.100 V vs Ag/AgCl (saturated KCl). The GEs with a "*b*" superscript are incremented by 0.020 V per generator per set while the CEs are held constant at – 0.100 V vs Ag/AgCl (saturated KCl).

6.6. Figure S6: CA data for Snapshot RCV from Potential Sets in Table 1 using 0.50 mM L-DOPA in aCSF

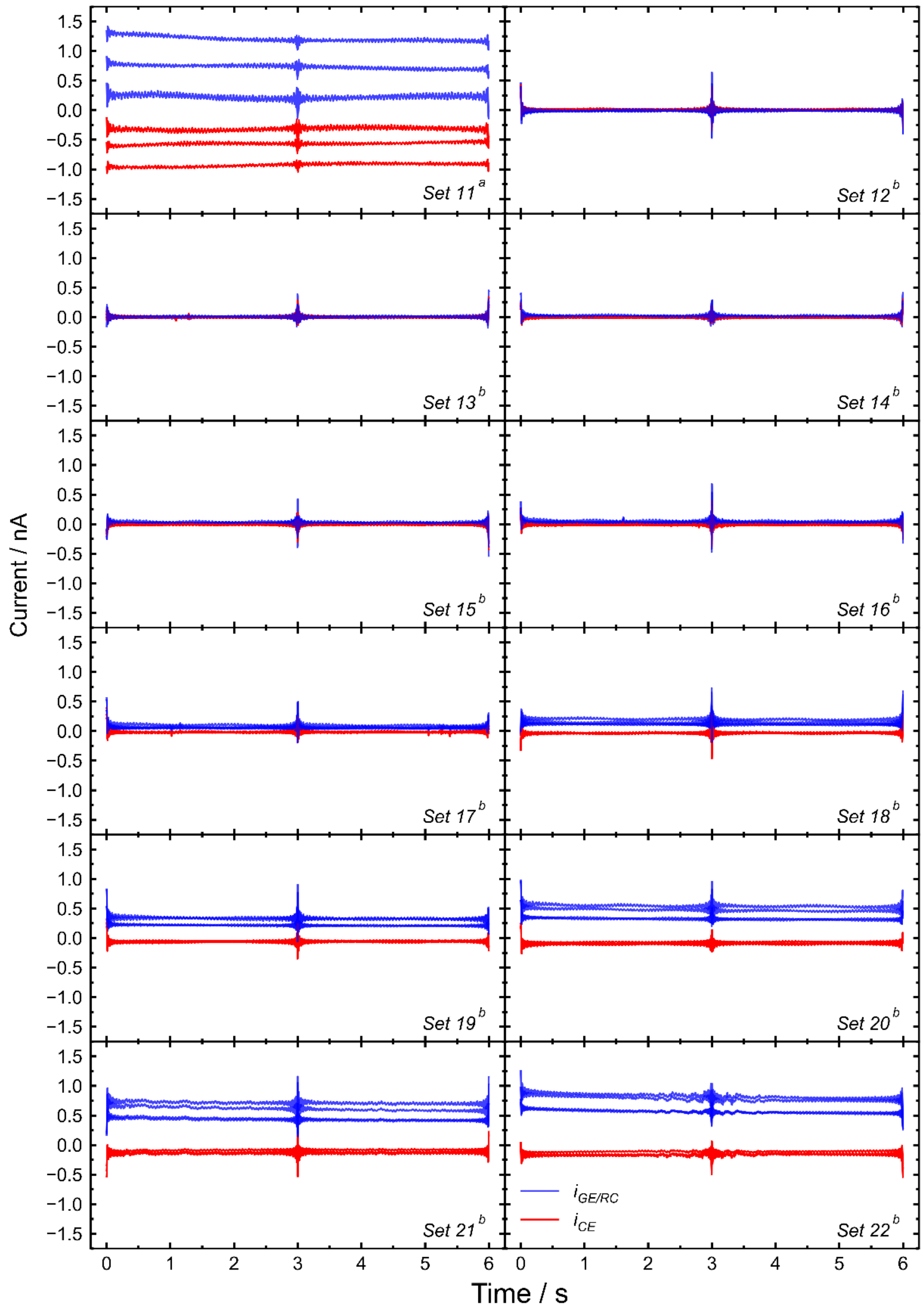

Figure S6. Raw snapshot RCV CA data for sets 11[a] – 22[b] listed in Table 1 of the main document for a solution of 0.50 mM 3,4-dihydroxyphenylalanine (L-DOPA) in aCSF. Generator (blue) and collector (red) electrodes are individually biased to the potentials listed in Table 1 and held constant

for 6 s with a 1 s quiet time. The GEs of set 11 with an "*a"* superscript are incremented by 0.060 V between electrodes while CEs are held constant at – 0.100 V vs Ag/AgCl (saturated KCl). The GEs with a "*b*" superscript are incremented by 0.020 V per generator per set while the CEs are held constant at – 0.100 V vs Ag/AgCl (saturated KCl).

6.7. Figure S7: CA data for Snapshot RCV from Potential Sets in Table 1 using 0.50 mM DOPAC in aCSF

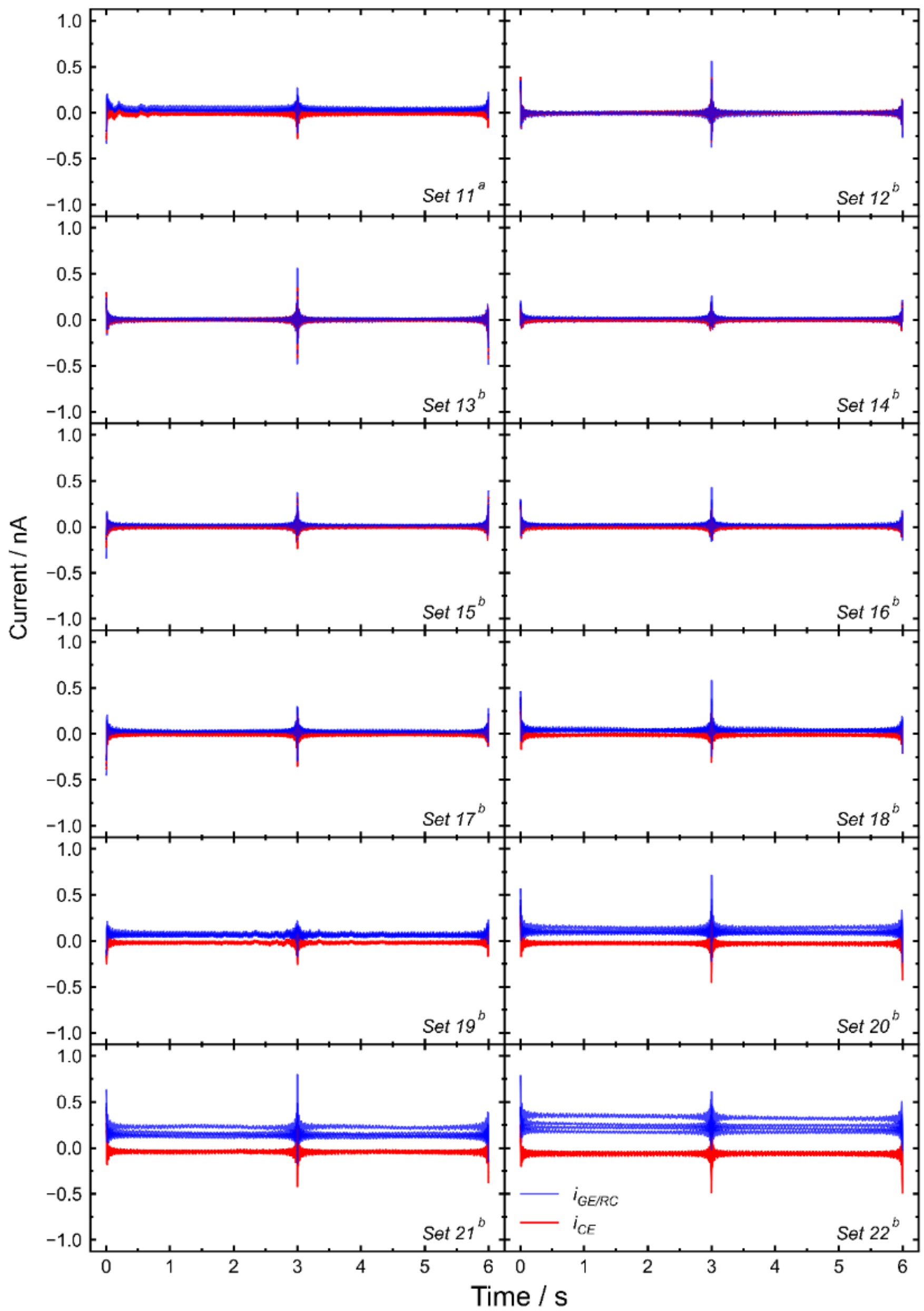

Figure S7. Raw snapshot RCV CA data for sets 11[a] – 22[b] listed in Table 1 of the main document for a solution of 0.50 mM 3,4-dihydroxyphenylacetic acid (DOPAC) in aCSF. Generator (blue) and collector (red) electrodes are individually biased to the potentials listed in Table 1 and held constant for 6 s with a 1 s quiet time. The GEs of set 11 with an "*a*" superscript are incremented

by 0.060 V between electrodes while CEs are held constant at – 0.100 V vs Ag/AgCl (saturated KCl). The GEs with a "*b*" superscript are incremented by 0.020 V per generator per set while the CEs are held constant at – 0.100 V vs Ag/AgCl (saturated KCl).

6.8. Figure S8: CA data for Snapshot RCV from Potential Sets in Table 1 using 0.50 mM AA in aCSF

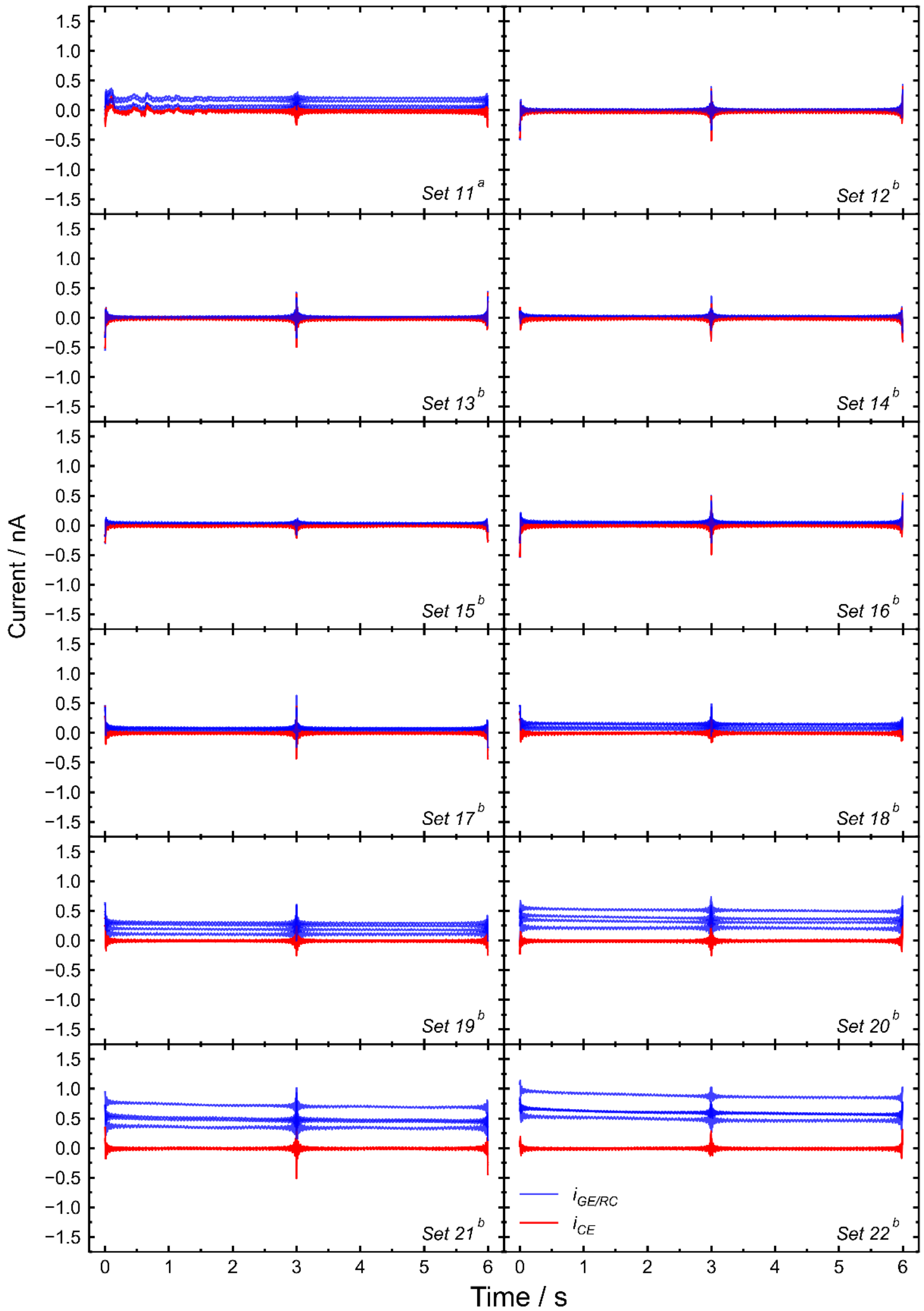

Figure S8. Raw snapshot RCV CA data for sets 11[a] – 22[b] listed in Table 1 of the main document for a solution of 0.50 mM ascorbic acid (AA) in aCSF. Generator (blue) and collector (red) electrodes are individually biased to the potentials listed in Table 1 and held constant for 6 s with

a 1 s quiet time. The GEs of set 11 with an "*a*" superscript are incremented by 0.060 V between electrodes while CEs are held constant at – 0.100 V vs Ag/AgCl (saturated KCl). The GEs with a "*b*" superscript are incremented by 0.020 V per generator per set while the CEs are held constant at -0.100 V vs Ag/AgCl (saturated KCl).

6.9. Figure S9. Overlay of GE and CE currents for conventional RCV and snapshot RCV (including the flanking GEs) for DA, L-DOPA, DOPAC and AA in aCSF

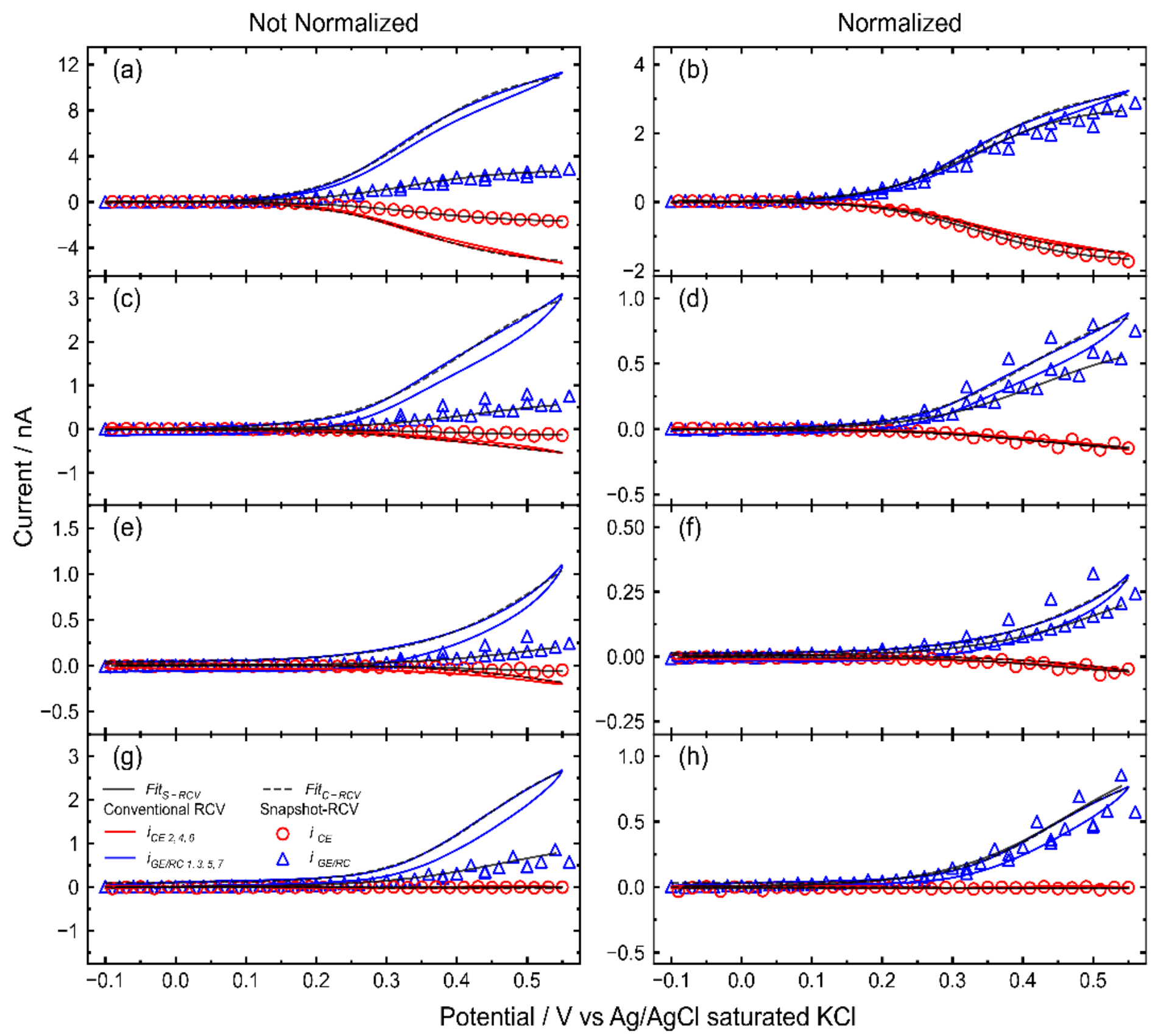

Figure S9. Overlay of GE and CE currents for conventional RCV and snapshot RCV (including the flanking GEs) in aCSF solution containing 0.50 mM DA (a-b), 0.50 mM L-DOPA (c-d), 0.50 mM DOPAC (e-f), and 0.50 mM AA (g-h). Conventional RCV was performed by sweeping the shorted GEs from – 0.100 V to + 0.550 V and back at 0.020 V s^{-1} and holding the potential of the shorted CEs at -0.100 V. Both the resulting GE current (blue curves) and CE currents (red curves) are plotted as a function of the GE potential. Snapshot RCV was performed in sets, with each GE and CE stepped individually and simultaneously to the potentials listed in Table 1 of the main document. Current at each electrode was averaged from 4 to 5 s (N=500). Each averaged GE current (blue triangles) is plotted as a function of the potential that was applied to that electrode.

Each averaged CE current (red circles) is plotted as a function of the potential that is halfway between the potentials applied to its adjacent generators. Sigmoidal fits (Conventional RCV, $Fit_{C\text{-}RCV}$, dashed black curves; Snapshot RCV, $Fit_{S\text{-}RCV}$, black solid curves) to the GE and CE responses are also shown. In (a, c, e, g) the data are not normalized. In (b, d, f, h) the conventional RCV responses have been normalized by 3.5, the number of electrode pairs.

7. References

(1) Peltola, E.; Sainio, S.; Holt, K. B.; Palomäki, T.; Koskinen, J.; Laurila, T. Electrochemical Fouling of Dopamine and Recovery of Carbon Electrodes. Analytical Chemistry 2018, 90 (2), 1408–1416. DOI: 10.1021/acs.analchem.7b04793.

(2) Schindler, S.; Bechtold, T. Mechanistic insights into the electrochemical oxidation of dopamine by cyclic voltammetry. *Journal of Electroanalytical Chemistry* **2019**, *836*, 94–101. DOI: 10.1016/j.jelechem.2019.01.069.

(3) Amatore, C. A.; Fosset, B.; Deakin, M. R.; Wightman, R. M. Electrochemical kinetics at microelectrodes: Part III. Equivalency between band and hemicylinder electrodes. *Journal of Electroanalytical Chemistry and Interfacial Electrochemistry* **1987**, *225* (1), 33–48. DOI: 10.1016/0022-0728(87)80003-7.

(4) Deakin, M. R.; Wightman, R. M.; Amatore, C. A. Electrochemical kinetics at microelectrodes: Part II. Cyclic voltammetry at band electrodes. *Journal of Electroanalytical Chemistry and Interfacial Electrochemistry* **1986**, *215* (1), 49–61. DOI: 10.1016/0022-0728(86)87004-8.

(5) Amatore, C. A.; Deakin, M. R.; Wightman, M. Electrochemical kinetics at microelectrodes Part 1. Quasi-reversible electron transfer at cylinders. *Journal of Electroanalytical Chemistry and Interfacial Electrochemistry* **1986**, *206* (1), 23–36. DOI: 10.1016/0022-0728(86)90253-6.

(6) Bieniasz, L. K. Theory of Potential Step Chronoamperometry at a Microband Electrode: Complete Explicit Semi-Analytical Formulae for the Faradaic Current Density and the Faradaic Current. *Electrochim. Acta* **2015**, *178*, 25–33. DOI: 10.1016/j.electacta.2015.07.040.

(7) Bieniasz, L. K. Highly accurate, inexpensive procedures for computing theoretical chronoamperometric currents at single straight electrode edges and at single microband electrodes. *J. Electroanal. Chem.* **2016**, *760*, 71–79. DOI: 10.1016/j.jelechem.2015.11.040.

(8) Licht, S.; Cammarata, V.; Wrighton, M. S. Direct measurements of the physical diffusion of redox active species: microelectrochemical experiments and their simulation. *The Journal of Physical Chemistry* **1990**, *94* (15), 6133–6140. DOI: 10.1021/j100378a090.

(9) Bard, A. J.; Crayston, J. A.; Kittlesen, G. P.; Varco Shea, T.; Wrighton, M. S. Digital simulation of the measured electrochemical response of reversible redox couples at microelectrode arrays: consequences arising from closely spaced ultramicroelectrodes. *Anal. Chem.* **1986**, *58* (11), 2321–2331. DOI: 10.1021/ac00124a045.

(10) Abrego Tello, M. A.; Lotfi Marchoubeh, M.; Fritsch, I. In Situ and 2D and 3D in Silico Redox Cycling Studies for Design Optimization of Coplanar Arrays of Microband Electrodes in a 70 μm × 100 μm Electroactive Footprint. *Journal of The Electrochemical Society* **2024**, *171* (6), 066512. DOI: 10.1149/1945-7111/ad5409.

(11) Mirkin, M. V.; Bard, A. J. Simple analysis of quasi-reversible steady-state voltammograms. *Anal. Chem.* **1992**, *64* (19), 2293–2302. DOI: 10.1021/ac00043a020.

(12) Lotfi Marchoubeh, M.; Cobb, S. J.; Abrego Tello, M.; Hu, M.; Jaquins-Gerstl, A.; Robbins, E. M.; Macpherson, J. V.; Michael, A. C.; Fritsch, I. Miniaturized probe on polymer SU-8 with array of individually addressable microelectrodes for electrochemical analysis in neural and other biological tissues. *Analytical and Bioanalytical Chemistry* **2021**, *413* (27), 6777–6791. DOI: 10.1007/s00216-021-03327-2.
